# The *sps* Genes Encode an Original Legionaminic Acid Pathway Required for Crust Assembly in Bacillus subtilis

**DOI:** 10.1128/mBio.01153-20

**Published:** 2020-08-18

**Authors:** Thomas Dubois, Frederic Krzewinski, Nao Yamakawa, Christelle Lemy, Audrey Hamiot, Loïc Brunet, Anne-Sophie Lacoste, Yuryi Knirel, Yann Guerardel, Christine Faille

**Affiliations:** a University Lille, CNRS, INRAE, Centrale Lille, UMR 8207—UMET—Unité Matériaux et Transformations, Lille, France; b University Lille, CNRS, UMR 8576—UGSF—Unité de Glycobiologie Structurale et Fonctionnelle, Lille, France; c University Lille, CNRS, INSERM, CHU Lille, Institut Pasteur de Lille, US 41—UMS 2014—PLBS, Lille, France; d N.D. Zelinsky Institute of Organic Chemistry, Russian Academy of Sciences, Moscow, Russia; University of Delaware

**Keywords:** *Bacillus subtilis*, bacterial adhesion, crust, legionaminic acid, nonulosonic acid, spores

## Abstract

The crust is the outermost spore layer of most *Bacillus* strains devoid of an exosporium. This outermost layer, composed of both proteins and carbohydrates, plays a major role in the adhesion and spreading of spores into the environment. Recent studies have identified several crust proteins and have provided insights about their organization at the spore surface. However, although carbohydrates are known to participate in adhesion, little is known about their composition, structure, and localization. In this study, we showed that the spore surface of Bacillus subtilis is covered with legionaminic acid (Leg), a nine-carbon backbone nonulosonic acid known to decorate the flagellin of the human pathogens Helicobacter pylori and Campylobacter jejuni. We demonstrated that the *spsC*, *spsD*, *spsE*, *spsG*, and *spsM* genes of Bacillus subtilis are required for Leg biosynthesis during sporulation, while the *spsF* gene is required for Leg transfer from the mother cell to the surface of the forespore. We also characterized the activity of SpsM and highlighted an original Leg biosynthesis pathway in B. subtilis. Finally, we demonstrated that Leg is required for the assembly of the crust around the spores, and we showed that in the absence of Leg, spores were more adherent to stainless steel probably because of their reduced hydrophilicity and charge.

## Abstract (precis)

*Bacillus* species are a major economic and food safety concern of the food industry because of their food spoilage-causing capability and persistence. Their persistence is mainly due to their ability to form highly resistant spores adhering to the surfaces of industrial equipment. Spores of the Bacillus subtilis group are surrounded by the crust, a superficial layer which plays a key role in their adhesion properties. However, knowledge of the composition and structure of this layer remains incomplete. Here, for the first time, we identified a nonulosonic acid (Leg) at the surfaces of bacterial spores (B. subtilis). We uncovered a novel Leg biosynthesis pathway, and we demonstrated that Leg is required for proper crust assembly. This work contributes to the description of the structure and composition of *Bacillus* spores which has been under way for decades, and it provides keys to understanding the importance of carbohydrates in *Bacillus* adhesion and persistence in the food industry.

## INTRODUCTION

The *Bacillaceae* are able to engage in a process of cellular differentiation called sporulation, which results in the formation of a mature spore capable of surviving adverse environmental conditions and of being dispersed into the environment. Because of these properties, *Bacillus* spores are ubiquitous in the environment and contaminate many raw materials of the food industry, e.g., in the dairy industry, causing heavy economic losses and a health risk to consumers (1).

The *Bacillus* spore consists of a series of concentric layers. Its core contains highly condensed DNA wrapped around proteins called “small soluble acid proteins,” which protect the DNA from physicochemical stresses. The core is surrounded by an inner membrane and a germ cell wall that are themselves covered by a spore-specific peptidoglycan called cortex (2, 3). The cortex is surrounded by an outer membrane, which is itself surrounded by the three-layered coat, composed of at least 80 different proteins (4). The coat proteins are recruited and assembled by morphogenetic proteins, with at least one morphogenetic protein per coat layer: SpoIVA for the basement layer, SafA for the inner coat, and CotE for the outer coat (5–8). In Bacillus subtilis and most of the *Bacillus* strains producing spores devoid of an exosporium, the outermost layer of spores is the crust (9). It is made of proteins and carbohydrates, and it plays a major role in the surface and adhesion properties of spores (9). Thus, the mechanical removal of the crust renders the spore less hydrophilic and more adherent to stainless steel (9). Therefore, the presence or absence of this outermost layer could determine the spreading properties of B. subtilis spores and their resistance to the cleaning-in-place procedures used in food industries (1, 10).

At least six proteins have been identified as constituents of the crust: CotV, CotW, CotX, CotY, CotZ, and CgeA (11–15). The crust structure is mainly provided by the CotV, CotX, and CotY proteins, among which, CotY would be the major structural component (15). Interestingly, CotV and CotX share homologue domains and putative *N*-glycosylation motifs, which make these proteins candidates for glycosylation (15). McKenney and colleagues suggested that CotX, CotY, and CotZ are the crust morphogenetic proteins (12), and recent studies have confirmed that these proteins play a major role in crust assembly (14, 15). CotZ could be the main morphogenetic protein, since it is involved in the proper localization of most of the crust proteins (14). CotV could also be involved in the crust assembly by propagating the crust structure from the polar cap-like structure to the middle part of the spore (15). CotZ, which required CotE and CotO but none of the crust proteins for localization, seems to have an important anchoring function for the crust in addition to its assembly function (15). CotW plays a role in maintaining the structural integrity of the crust and might play an anchoring role at the interface of crust and coat (14, 15). Finally, transmission electron microscopy (TEM) experiments showed that the crust of the *cotX* and *cgeA* mutants is assembled, but only loosely attached to spores, thus suggesting that CgeA and CotX play roles in the interaction of the crust with the coat (14).

While the crust protein interaction network begins to be better described, the localization, nature, and structure of the glycans on the spore surface remain mostly unknown. It was shown that the spore surface of B. subtilis contains at least rhamnose, galactose, quinovose, glucosamine, and muramic lactam (9, 16). Although the structure of the glycans composed of these monosaccharides is unknown, it was suggested that the spore surface contains at least two different glycans: one associated with the outer coat and at least one other linked to the crust (15, 17). The *spsM*, *spsABCDEFGIJKL*, *yfnHGFED*, *ytdA-ytcABC*, and *cgeAB-cgeCDE* genes were identified as participating in the morphology and the surface properties of the crust (15–18). Mutations in the *sps* genes result in the production of less hydrophilic spores, devoid of perceivable halo after negative staining with India ink, thus suggesting these genes play a role in spore surface glycosylation (16–18). Contrastingly, mutations in each of the *yfnHGFED* genes do not affect the spore hydrophilicity but modify the crust structure and expand the glycan layer (15, 17). Finally, spores of a *cgeD* mutant strain are less hydrophilic and the superficial saccharide layer is expanded, just as it is with the mutants of the *yfnHGFED* genes (17). These data resulted in the identification of some of the enzyme-encoding genes that synthesize the superficial spore glycans. However, to date, both these genes’ functions and the biosynthetic pathway in which they are involved remain unknown, with the notable exception of the *spsIJKL* genes, known to encode the biosynthesis pathway converting d-glucose-1-phosphate to dTDP-l-rhamnose (19–23).

Here, we sought to define the functions of the *sps* genes and their roles in crust biosynthesis. Through an approach that combines genetic and biochemical methods, we demonstrated that six of the *sps* genes encode an original CMP-legionaminic acid (CMP-Leg) pathway, and we showed that legionaminic acid (Leg) is required for proper crust assembly. This work contributes to a better understanding of the outermost spore layer composition and structure that influence the adhesion and spreading of B. subtilis in the environment.

## RESULTS

### The *sps* genes contribute to the surface and adhesion properties of Bacillus subtilis spores.

**FIG 1:**
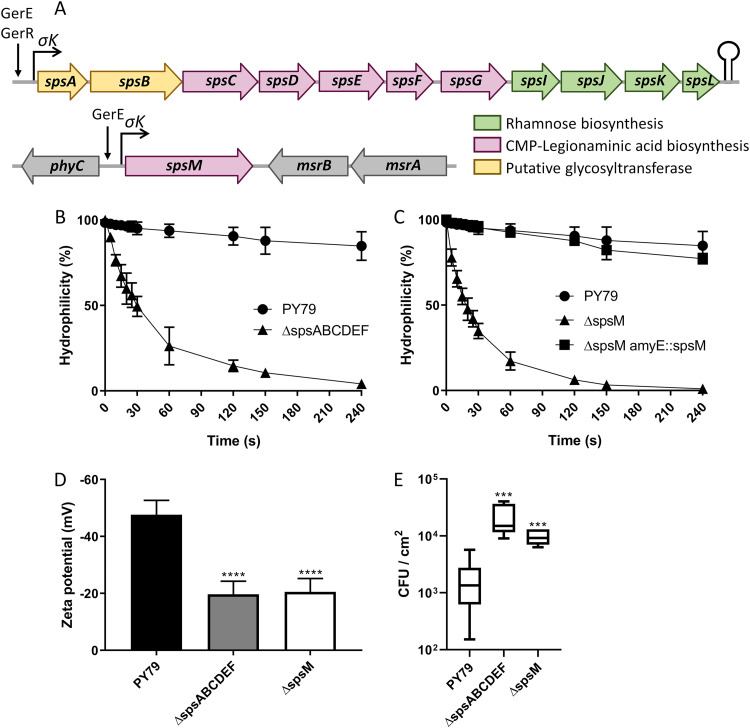
The *sps* genes participate in the surface and adhesion properties of B. subtilis spores. (A) Schematic representation of the *spsABCDEFGIJKL* and *spsM* genes in B. subtilis PY79. Transcriptional activators and promoters are shown with arrows and broken arrows, respectively. Potential stem-loop structure is indicated with a lollipop. The annotation of the *sps* genes is presented in Table S1 in the supplemental material. (B) Surface hydrophilicity of spores of the PY79 and PY79 Δ*spsABCDEF* (Δ*spsABCDEF*) strains evaluated by MATH assays. (C) Surface hydrophilicity of spores of the PY79, PY79 Δ*spsM* (Δ*spsM*), and PY79 Δ*spsM amyE*::*spsM* (Δ*spsM amyE*::*spsM*) strains. (D) Surface charge of spores evaluated by zetametry assays. (E) Adhesion of spores to stainless steel coupons. The results are expressed in CFU per square centimeter of stainless steel. The data are averages from at least three independent experiments performed with spores prepared independently. The error bars represent the standard deviations (SDs) of the means. ***, *P ≤ *0.001; ****, *P ≤ *0.0001 for Δ versus PY79 by Mann-Whitney test.

The *sps* genes are distributed in two loci on the B. subtilis chromosome (Fig. 1A). The first locus contains the *spsABCDEFGIJKL* genes and the second contains the *spsM* gene. The two loci are transcribed in the mother cell from σ^K^-dependent promoters located upstream from the *spsA* and *spsM* genes (16, 18, 24–26). This transcriptional regulation indicates that the products of the *sps* genes are produced concomitantly in the mother cell during the late stage of sporulation. To evaluate the role of the *sps* genes, mutant strains of the *spsABCDEF* and *spsM* genes were constructed in the B. subtilis PY79 strain (subsequently named PY79). Spores were produced, and their surface properties were characterized. The hydrophilicity of the spores was assessed through microbial-adhesion-to-hydrocarbons (MATH) (Fig. 1B and C). Most PY79 spores remained in the aqueous phase throughout the MATH experiment, which reflected a marked hydrophilic character. In contrast, the percentage of spores in the aqueous layer for the Δ*spsABCDEF* and Δ*spsM* mutant strains decreased over time and tended toward zero after 240 s of agitation. These results indicate that the Δ*spsABCDEF* and Δ*spsM* mutant strains are less hydrophilic than the PY79 strain, as is consistent with previous studies (16–18). In addition, the complementation of the Δ*spsM* mutant strain restored the hydrophilic properties of the PY79 strain (Fig. 1C). This indicates that the phenotype observed with the Δ*spsM* mutant strain is not due to a polar effect of the mutation on flanking genes expression. The overall spore charge at neutral pH was then evaluated using zetametry (Fig. 1D). The zeta potentials of the Δ*spsABCDEF* and Δ*spsM* spores were around −20 mV, indicating that they are less charged than the PY79 spores (−46.4 mV). These data demonstrate that the *spsABCDEF* and *spsM* gene products contribute to the hydrophilicity and the negative charge of spores that may lead to changes in spore adhesion properties. The role of the *sps* genes in the adhesion of spores to stainless steel coupons was therefore examined (Fig. 1E). With the PY79 strain, an average of 1.8 × 10^3^ spores adhered to the stainless-steel coupons, while 12 and 5 times more spores adhered with the Δ*spsABCDEF* and Δ*spsM* mutant strains, respectively, thus indicating that the *sps* gene products influence spore adhesion properties.

10.1128/mBio.01153-20.9 TABLE S1 Annotation of the *sps* genes and RaptorX predictions. The putative function of the *sps* gene products are extracted from the MaGe genome browser (http://www.genoscope.cns.fr/). The structures from the PDB database that are the most similar to the RaptorX predicted structures of the Sps proteins are shown in the table. Download Table S1, DOCX file, 0.1 MB. Copyright © 2020 Dubois et al. 2020 Dubois et al. This content is distributed under the terms of the Creative Commons Attribution 4.0 International license.

### The *sps* genes are required for crust assembly.

**FIG 2:**
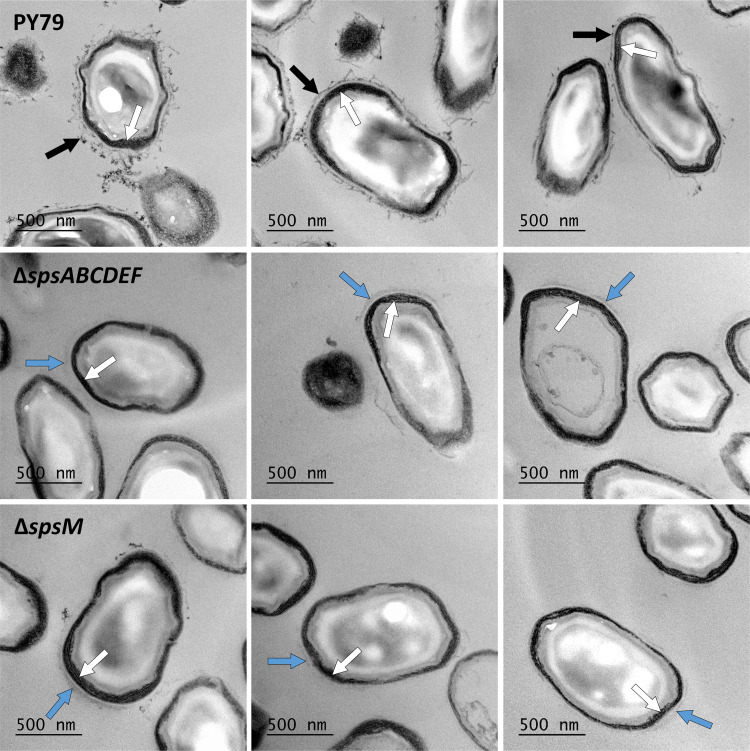
The *sps* genes are required for crust assembly. TEM images of spore sections after ruthenium red staining. The experiments were performed with the spores of the PY79, PY79 Δ*spsABCDEF* (Δ*spsABCDEF*), and PY79 Δ*spsM* (Δ*spsM*) strains. White arrows, coat layers; black arrows, crust; blue arrows, cap-like structures.

The surface and adhesion properties of spores of the Δ*spsABCDEF* and Δ*spsM* mutant strains were very similar to those of spores lacking the crust (see Fig. S1 in the supplemental material), which suggests that the *sps* genes are important for proper crust assembly. To test this assumption, the spore morphology of the PY79, Δ*spsABCDEF*, and Δ*spsM* strains was observed by TEM using spore sections contrasted with ruthenium red. This staining enables the observation of the carbohydrate-rich layer on the spore surface (27). The crust forms an exosporium-like structure that is clearly visible for PY79 spores (black arrows) (Fig. 2). This exosporium-like structure was no longer observed on the spore surfaces of the Δ*spsABCDEF* and Δ*spsM* mutant strains, thereby confirming that the *sps* genes are required for proper crust assembly. However, a loose low-contrast material was still visible, mostly at the spore poles (blue arrows). This could be reminiscent of a polar cap-like structure from which the crust propagates to the middle part of the spore. These observations suggest that the products of the *sps* genes participate in the propagation of the crust or in anchoring the crust to the outer coat.

10.1128/mBio.01153-20.1 FIG S1 Influence of the crust removal on the surface and adhesion properties of spores. Experiments were performed with spores of PY79 strain with crust (PY79) or crustless (PY79 −crust). The crust was removed using a French press as described in Materials and Methods. It was shown previously that the French press procedure does not damage the spore coat (9). (A) Surface hydrophilicity of spores evaluated by MATH assays (B) Surface charge of spores evaluated by zetametry assays. The error bars represent the SDs of the means. ****, *P ≤ *0.0001 for PY79 −crust versus PY79 by Mann-Whitney test. (C) Evaluation of spore adhesion on stainless steel coupons. Drops of spores (5 μl at 10^9^ CFU/ml) were deposited on stainless steel coupons and dried for 30 min at room temperature. The coupons were then washed three times in a water bath by immersion and emersion to remove loosely adhering spores. Pictures were taken before and after the washing step. Download FIG S1, TIF file, 1.4 MB. Copyright © 2020 Dubois et al. 2020 Dubois et al. This content is distributed under the terms of the Creative Commons Attribution 4.0 International license.

### The *sps* genes participate in spore surface glycosylation.

The function of the enzymes encoded by the *sps* genes and the biosynthetic pathways in which these enzymes are involved have not been described. To better define the role of the *sps* genes, the amount of neutral sugars on the PY79 spore surface was measured. For this purpose, spores were mechanically treated with a French press to remove their surface fraction without damaging the spore coat (9). This surface fraction contains the crust proteins and the potentially associated carbohydrates as well as the constituents located between the outer coat proteins and the crust. The amount of neutral sugars in the surface fraction was then measured by the phenol-sulfuric method (28) (see Fig. S2). It was significantly lower in the surface fraction of the Δ*spsABCDEF* and Δ*spsM* mutant strains than in the PY79 strain. Knowing the putative function of the *sps* genes (Table S1), this might indicate that the *spsABCDEF* and *spsM* genes are involved in the production or the attachment of one or several neutral sugars on the spore surface. However, given that the Δ*spsABCDEF* and Δ*spsM* mutant strains lack most of the crust (Fig. 2), it is also possible that the neutral sugar decrease was an indirect consequence of the crust assembly defect, such as the loss of one or several crust-linked glycan(s).

10.1128/mBio.01153-20.2 FIG S2 Quantification of neutral sugars in the surface fraction of spores by the phenol-sulfuric method. The results were standardized by the OD_600_ of the spore preparations. The error bars represent the SDs of the means. ***, *P ≤ *0.001 for Δ versus PY79 by *t* test. Download FIG S2, TIF file, 0.6 MB. Copyright © 2020 Dubois et al. 2020 Dubois et al. This content is distributed under the terms of the Creative Commons Attribution 4.0 International license.

### The surface fraction of spores contains legionaminic acid.

**FIG 3:**
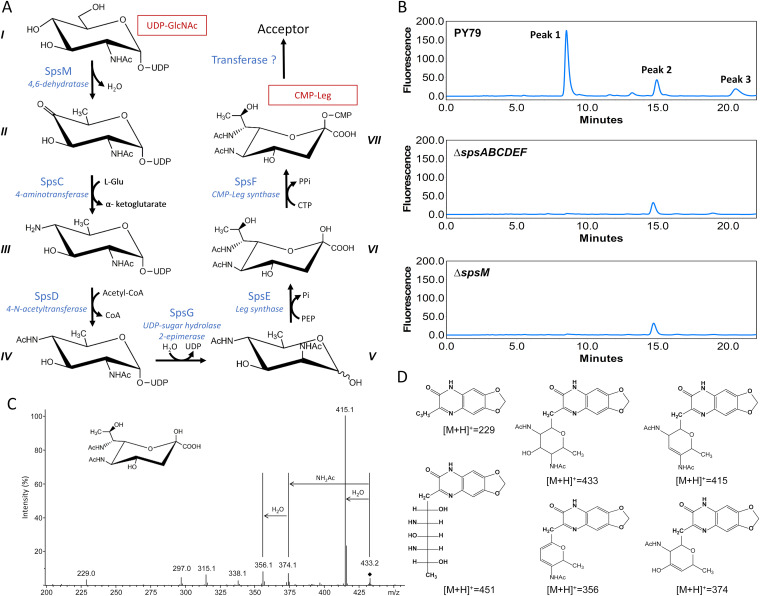
The *sps* genes encode a pathway required for legionaminic acid addition on the spore surface of B. subtilis. (A) Predicted Leg pathway in B. subtilis: I, UDP-*N*-acetyl-α-d-glucosamine (UDP-GlcNAc); II, UDP-2-acetamido-2,6-dideoxy-α-*d-xylo*-hexose-4-ulose (UDP-4-keto-6-deoxy-GlcNAc); III, UDP-4-amino-4,6-dideoxy-*N*-acetyl-α-d-glucosamine (UDP-4-amino-6-deoxy-GlcNAc); IV, UDP-2,4-diacetamido-2,4,6-trideoxy-α-d-glucose (UDP-2,4-diNAc-6-deoxy-Glc); V, 2,4-diacetamido-2,4,6-trideoxy-d-mannose (2,4-diNAc-6-deoxy-Man); VI, 5,7-diacetamido-3,5,7,9-tetradeoxy-d-glycero-β-d-galacto-nonulosonic acid (legionaminic acid or Leg); VII, CMP-5,7-diacetamido-3,5,7,9-tetradeoxy-d-glycero-β-d-galacto-nonulosonic acid (CMP-legionaminic acid or CMP-Leg). (B) Chromatograms of the RP-HPLC-FL experiments performed on the surface fractions of spores of the PY79, PY79 Δ*spsABCDEF* (Δ*spsABCDEF*), and PY79 Δ*spsM* (Δ*spsM*) strains. The RP-HPLC-FL experiments were carried out on at least three independent surface fractions for each strain. One representative chromatogram is presented. (C) Identification by LC/ESI-MS^3^ of DMB-Leg5Ac7Ac. Representative MS^3^ spectra of DMB-Leg5Ac7Ac, [M+H]^+^ at *m/z* 451. The ion [M+H-18]^+^ at *m/z* 433 is indicated by the black diamond. (D) Structure of DMB ([M+H]^+^ = 229), DMB-Leg ([M+H]^+^ = 541), and of the major structure fragments ([M+H]^+^ = 433, [M+H]^+^ = 415.1, [M+H]^+^ = 374, [M+H]^+^ = 356).

Similar phenotypes being obtained with the Δ*spsABCDEF* and Δ*spsM* mutant strains, these genes probably participate in the same carbohydrate biosynthetic pathway. To better understand the role of the Sps proteins in the spore surface glycosylation, *in silico* analysis was performed using RaptorX software with the primary sequences of the SpsA-G and SpsM proteins (29). The RaptorX software predicts a tridimensional structure of a query sequence and compares this predicted structure to known protein structures from the Protein Data Bank. The outputs of this analysis are presented in Table S1 and they are further described in the discussion section. By using gene annotations and RaptorX predictions, a predictive biosynthetic pathway was constructed (Fig. 3A). This pathway is reminiscent of the pseudaminic acid (Pse) biosynthetic pathway of Helicobacter pylori and the Leg biosynthetic pathway of Campylobacter jejuni (30, 31). Pse and Leg are nine-carbon-backbone sialic acid analogs known to be crucial for flagellin glycosylation and virulence of C. jejuni and H. pylori (32–36). To ensure that the spore surface contains sialic acid-related compounds, the potential nonulosonic acids (NulOs) from the surface fraction of the PY79 strain were derivatized by 1,2-diamino-4,5-methylenedioxybenzene (DMB), a fluorogenic reagent that shows high specificity for NulOs, and analyzed by reverse-phase high-performance liquid chromatography coupled to fluorescence detector (RP-HPLC-FL) (Fig. 3B). RP-HPLC-FL analysis showed three peaks with retention times of ∼8.5 min (peak 1), 14.9 min (peak 2), and 20.5 min (peak 3), which were then analyzed in liquid chromatography mass spectrometry (LC-MS). Unlike in peaks 2 and 3, an MS signal at *m/z* 451 was identified in peak 1, corresponding to the [M+H]^+^ adduct of a compound with a molecular mass of 450.25 Da (data not shown). This signal corresponds to the expected mass of DMB-Leg, DMB-Pse, or one of their isomers that exhibits a [M+H]^+^ value at *m/z* 451 (37, 38). The identification was then confirmed by MS^3^ (Fig. 3C and D). MS^3^ analysis showed recurrent losses of H_2_O (*m/z* 433, *m/z* 415, and *m/z* 356) and *N*-acetyl groups (*m/z* 374), which agrees with the fragmentation pattern previously obtained with *N*-acetylneuraminic acid (Neu5Ac) and other NulOs (36, 37). Furthermore, the retention time of peak 1 perfectly matched that of the Leg standard (see Fig. S3), thus indicating that the surface fraction of PY79 spores contains Leg.

10.1128/mBio.01153-20.3 FIG S3 Chromatograms of the RP-HPLC-FL experiments performed on a mix of Leg and Pse standards (A) and the surface fraction of spores of the PY79 (B) and 168 (C) strains. To discriminate between the standards, a C_18_ Wakopak Handy ODS 4.6-mm by 250-mm column was used in this experiment. This column is longer than the one used in the other experiments, which explains the increase retention time of Leg (Fig. 3B, peak 1 and Fig. S3B). Download FIG S3, TIF file, 0.8 MB. Copyright © 2020 Dubois et al. 2020 Dubois et al. This content is distributed under the terms of the Creative Commons Attribution 4.0 International license.

### The *sps* genes expressed during the late stage of sporulation are required for legionaminic acid addition on the spore surface.

To determine whether the *sps* genes are involved in Leg biosynthesis, the surface fractions of the PY79, Δ*spsABCDEF*, and Δ*spsM* spores were analyzed by RP-HPLC-FL (Fig. 3B). Interestingly, Leg (peak 1) was undetectable in the surface fraction of the Δ*spsABCDEF* and Δ*spsM* mutant strains. These results demonstrate that *spsM* and at least one of the *spsABCDEF* genes are required for Leg biosynthesis or transfer to the spore surface. To study the kinetics of the *sps* genes transcription, a chromosomal transcriptional fusion was constructed by inserting the promoter region of the *spsA* gene fused to a promoterless *mCherry* gene into the *amyE* gene, and the resulting strain was designated PY79 *amyE*::P*_spsA_*-*mCherry*. The kinetics of *sps* genes expression was then determined in the Spo8 sporulation medium (see Fig. S4A). The PY79 *amyE*::P*_spsA_*-*mCherry* strain displayed *mCherry* fluorescence 6 h after the transition to the stationary phase (t6) and peaked at t8, which is in agreement with a previous study (18). Transcription of the *sps* genes is under the control of σ^K^, indicating that the Sps enzymes and their products are localized in the mother cell (16, 18, 24–26). To define the timing of Leg production in the mother cell, a kinetics analysis of Leg production was performed under the same growth conditions (Fig. S4B). Consistently with the transcription kinetics, the Leg was detectable in the PY79 strain at t8 and peaked at t10, approximately 2 h following the transcription peak. Together, these results indicate that Leg is produced in the mother cell of sporulating cells during the late stage of sporulation.

10.1128/mBio.01153-20.4 FIG S4 The *sps* genes are expressed during the late stage of sporulation. (A) Expression kinetics of the P_spsA_-*mCherry* transcriptional fusion in the PY79 strain. (B) Kinetics of Leg production in the mother cell of the PY79 strain. The amount of Leg was evaluated by RP-HPLC-FL. The results were standardized by the OD_600_ of the bacterial cultures. Growth for both kinetics were performed in Spo8 medium at 37°C. Time on the *x* axes is given relative to the transition to stationary phase. Error bars represent the SDs of the means. Download FIG S4, TIF file, 1.2 MB. Copyright © 2020 Dubois et al. 2020 Dubois et al. This content is distributed under the terms of the Creative Commons Attribution 4.0 International license.

### SpsM is the first enzyme of the legionaminic acid biosynthesis pathway.

**FIG 4:**
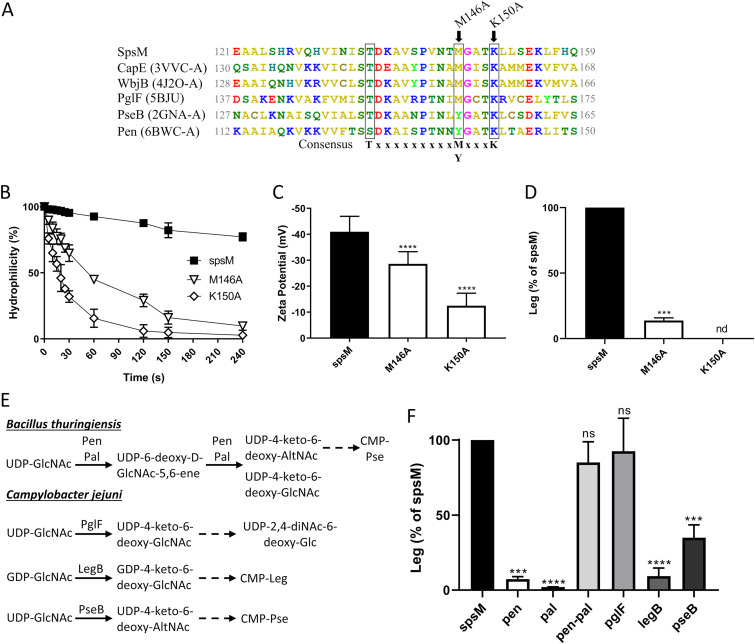
SpsM is a C-4/C-6 dehydratase using UDP-GlcNAc as a substrate. (A) Alignment of the protein sequence of SpsM with the protein sequences of CapE (Staphylococcus aureus), WbjB (Acinetobacter baumannii), PglF (Campylobacter jejuni), PseB (Helicobacter pylori), and Pen (Bacillus thuringiensis). The PDB number corresponding to each sequence is indicated in parentheses. The three conserved residues of the catalytic triad are black boxed. The arrows indicate residues modified by directed mutagenesis. The experiments presented in panels B, C, and D were carried out with PY79 Δ*spsM amyE*::*spsM* (*spsM*), PY79 Δ*spsM amyE*::*spsM* M146A (M146A), and PY79 Δ*spsM amyE*::*spsM* K150A (K150A) strains. (B) Surface hydrophilicity of spores evaluated by MATH assays. (C) Surface charge of spores evaluated by zetametry assays. ****, *P ≤ *0.0001 for M146A or K150A versus *spsM* by Mann-Whitney test. (D) Relative amounts of Leg in the surface fractions of spores measured by RP-HPLC-FL. The results are relative to the amount of Leg of the PY79 Δ*spsM amyE*::*spsM* strain. nd, not detectable; ***, *P ≤ *0.001 for M146A or K150A versus *spsM* by Welch’s *t* test. (E) Enzymatic reactions catalyzed by Pen-Pal, PglF, LegB, and PseB in B. thuringiensis and C. jejuni. The solid arrows represent the enzymatic reactions catalyzed by the enzymes. The dotted arrows represent the remaining parts of the pathways and point to the final products. (F) Relative amounts of Leg in the mother cells of the PY79 Δ*spsM amyE*::*spsM* (*spsM*), PY79 Δ*spsM amyE*::*pen* (*pen*), PY79 Δ*spsM amyE*::*pal* (*pal*), PY79 Δ*spsM amyE*::*pen-pal* (*pen-pal*), PY79 Δ*spsM amyE*::*pglF* (*pglF*), PY79 Δ*spsM amyE*::*legB* (*legB*), and PY79 Δ*spsM amyE*::*pseB* (*pseB*) strains measured at t10 by RP-HPLC-FL. The results are relative to the amount of Leg in the PY79 Δ*spsM amyE*::*spsM* strain. ns, not significant; ***, *P ≤ *0.001; ****, *P ≤ *0.0001 for *pen*, *pal*, *pen-pal*, *pglF*, *legB*, or *pseB* versus *spsM* by Welch’s *t* test. The error bars represent the SDs of the means.

The predictive three-dimensional structure of SpsM is close to that of the enzymes CapE, WbjB, and PseB (Table S1). These enzymes catalyze the C-4/C-6 dehydration and C-5 epimerization of the UDP-GlcNAc to produce UDP-4-keto-6-deoxy-AltNAc, and they share a conserved catalytic domain with a distinctive T-(x)_9_-M/Y-(x)_3_-K catalytic triad (32, 39–41). In Staphylococcus aureus, the replacement of the methionine by an alanine in the catalytic triad of CapE causes a partial activity loss for this enzyme (42). To determine whether SpsM also shares this catalytic domain, a protein sequence alignment was performed (Fig. 4A). This analysis identified, in the SpsM protein sequence, a T-(x)_9_-M-(x)_3_-K catalytic triad in a well-conserved putative catalytic domain. Mutations were introduced into the coding sequence of the *spsM* gene by directed mutagenesis to replace methionine 146 or lysine 150 with an alanine (Fig. 4A). Both mutations caused a decrease in the hydrophilicity and global spore charge (Fig. 4B and C). In addition, M146A and K150A mutations led to a partial and a total loss of Leg in the surface fraction of spores (Fig. 4D). These results indicate that the M146 and K150 amino acids are in the SpsM catalytic site and are required for full SpsM activity. CapE, WbjB, PglF, PseB, and Pen-Pal share C-4/C-6 dehydratase activity and use a nucleotide-activated GlcNAc as the substrate (32, 39–41, 43), thus suggesting that SpsM shares these properties. However, the activity of these enzymes slightly varies from one to the other. For example, PglF only has a C-4/C-6 dehydratase activity, while PseB has an additional C-5 epimerase activity (40) (Fig. 4E). To better define SpsM activity, the *pen* or *pal* genes of Bacillus thuringiensis ATCC 35646 and the *pseB*, *legB*, and *pglF* genes of Campylobacter jejuni NCTC11168 were introduced downstream of the *spsM* promoter into the *amyE* locus of the PY79 Δ*spsM* mutant strain. The enzymatic reactions catalyzed by Pen-Pal, PglF, LegB, and PseB are summarized in Fig. 4E (30, 32, 40, 43). Then, the relative amounts of Leg in the mother cells of the Δ*spsM amyE*::P_spsM_-*pen*, Δ*spsM amyE*::P_spsM_-*pal*, Δ*spsM amyE*::P_spsM_-*pen*-*pal*, Δ*spsM amyE*::P_spsM_-*pseB*, Δ*spsM amyE*::P_spsM_-*pglF*, and Δ*spsM amyE*::P_spsM_-*legB* strains were evaluated at t10 by RP-HPLC-FL (Fig. 4F). The addition of the *pen*, *pal*, *legB*, or *pseB* gene to the Δ*spsM* mutant strain did not restore, or only partially restored, Leg production in the mother cells. Contrastingly, the addition of the *pen*-*pal* or *pglF* gene fully restored Leg production, thus indicating that SpsM catalyzes the C-4/C-6 dehydration of the UDP-GlcNAc to produce UDP-4-keto-6-deoxy-GlcNAc. Together, these results also demonstrate that SpsM is the first enzyme of the CMP-Leg pathway in B. subtilis (Fig. 3A).

### *spsC*, *spsD*, *spsE*, and *spsG* are required for Leg biosynthesis, while *spsF* is required for Leg transfer to the forespore surface.

**FIG 5:**
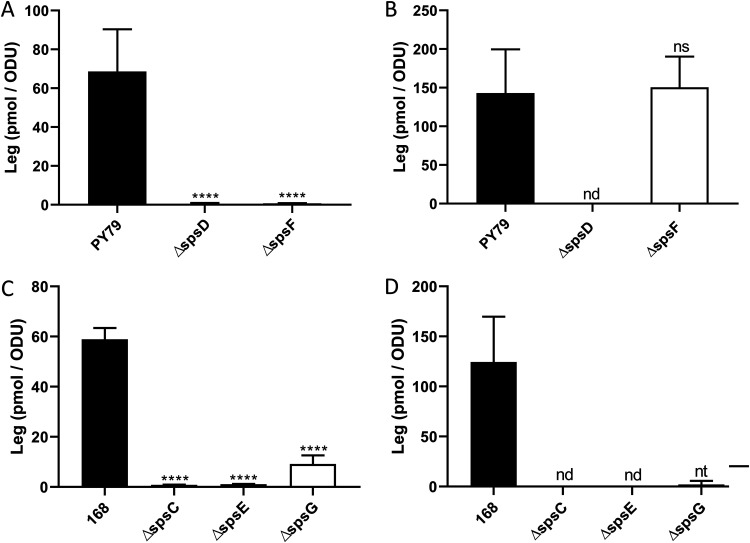
The *spsC*, *spsD*, *spsE*, and *spsG* genes are required for Leg biosynthesis, while *spsF* is required for Leg transfer to the forespore surface. The amounts of Leg were measured in the surface fractions of spores (A and C) and the mother cells of sporulating B. subtilis cells at t10 (B and D) by RP-HPLC-FL. The experiments were performed with PY79, PY79 Δ*spsD* (Δ*spsD*), and PY79 Δ*spsF* (Δ*spsF*) strains (A and B) or 168, 168 Δ*spsC* (Δ*spsC*), 168 Δ*spsE* (Δ*spsE*), and 168 Δ*spsG* (Δ*spsG*) strains (C and D). The results were standardized by the OD_600_ of the spore preparations (A and C) or the OD_600_ of the cultures at t10 (B and D). ns, not significant; nd, not detected; nt, not statistically tested because the data set did not pass the Shapiro-Wilk normality test (two values of three were equal to zero). The error bars represent the SDs of the means. ****, *P ≤ *0.0001 for Δ versus PY79 or 168 by Welch’s *t* test.

In the CMP-Leg biosynthesis pathway of B. subtilis proposed in Fig. 3A, the SpsM product is successively processed by SpsC, SpsD, SpsG, SpsE, and SpsF. It was shown above that one or more of the *spsABCDEF* genes are involved in Leg biosynthesis or transfer to the forespore surface (Fig. 3B). However, the involvement of each of these genes in the CMP-Leg pathway remained to be verified. To check the validity of the CMP-Leg pathway proposed in Fig. 3A, mutants of the *spsD* and *spsF* genes were constructed in the PY79 strain. The *spsD* and *spsF* genes encode a putative acetyltransferase and a putative cytidylyltransferase, respectively (Table S1). The spores of both mutants were less hydrophilic and negatively charged than those of the PY79 strain (see Fig. S5A, B, and E). The complementation of the Δ*spsD* mutant strain restored the hydrophilic properties of spores, while the complementation of the Δ*spsF* mutant strain did not. The lack of complementation of the Δ*spsF* mutant strain is discussed in Text S1. The surface fraction of spore was extracted and the Leg amount quantified (Fig. 5A). In parallel, the amounts of Leg in the mother cells of the PY79, Δ*spsD*, and Δ*spsF* strains were evaluated at t10 (Fig. 5B). Leg was absent from the surface fractions of the Δ*spsD* and Δ*spsF* mutant strains and from the mother cell of the Δ*spsD* mutant strain during sporulation. In contrast, the amount of Leg measured in the mother cell of the Δ*spsF* mutant strain was comparable to that of the PY79 strain. These results demonstrate that the *spsD* gene is required for Leg production, while the *spsF* gene is required for Leg transfer from the mother cell to the forespore. To test whether the *spsC*, *spsE*, and *spsG* genes are also required for Leg biosynthesis, we used the Δ*spsC*, Δ*spsE*, and Δ*spsG* mutants constructed in B. subtilis 168 strain (44). The SpsC, SpsE, and SpsG proteins of the 168 strain shared 100% of identity with the SpsC, SpsE, and SpsG proteins of the PY79 strain. In addition, the amount of Leg in the spore surface fraction of the 168 strain was similar to that of the PY79 strain (Fig. 5A and C and Fig. S3). Leg was absent from the spore surface fraction of the Δ*spsC* and Δ*spsE* mutant strains, and the Leg amount was strongly reduced in the Δ*spsG* mutant strain (Fig. 5C). Moreover, the quantification of Leg in the mother cells of the Δ*spsC*, Δ*spsE*, and Δ*spsG* mutant strains did not detect significant amounts of Leg (Fig. 5D). These data demonstrate that *spsC*, *spsE*, and *spsG* genes are required for Leg biosynthesis and are in accordance with the CMP-Leg pathway proposed in Fig. 3A.

10.1128/mBio.01153-20.5 FIG S5 Surface properties of spores of the *sps* mutants. Surface hydrophilicity of spores of the PY79 Δ*spsD* (Δ*spsD*) and PY79 Δ*spsD amyE*::*spsD* (Δ*spsD amyE*::*spsD*) (A), PY79 Δ*spsF* (Δ*spsF*) and PY79 Δ*spsF amyE*::*spsF* (Δ*spsF amyE*::*spsF*) (B), PY79 Δ*spsA* (Δ*spsA*) and PY79 Δ*spsA amyE*::*spsA* (Δ*spsA amyE*::*spsA*) (C), and PY79 Δ*spsB* (Δ*spsB*) and B. subtilis PY79 Δ*spsB amyE*::*spsB* (Δ*spsB amyE*::*spsB*) (D) strains evaluated by MATH assays. (E) Surface charge of spores evaluated by zetametry assays. The error bars represent the SDs of the means. ****, *P ≤ *0.0001 for Δ versus PY79 by Mann-Whitney test. Download FIG S5, TIF file, 2.4 MB. Copyright © 2020 Dubois et al. 2020 Dubois et al. This content is distributed under the terms of the Creative Commons Attribution 4.0 International license.

10.1128/mBio.01153-20.7 TEXT S1 Comment on the complementation of the Δ*spsF* mutant strain. Download Text S1, DOCX file, 0.1 MB. Copyright © 2020 Dubois et al. 2020 Dubois et al. This content is distributed under the terms of the Creative Commons Attribution 4.0 International license.

### SpsA and SpsB are not the transferase of the CMP-Leg.

**FIG 6:**
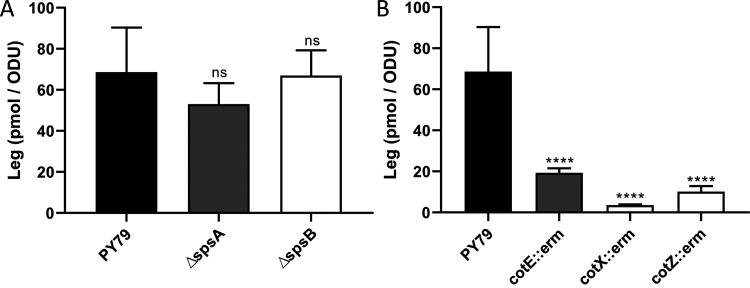
Legionaminic acid is linked to the crust. The amounts of Leg were measured in the surface fractions of spores by RP-HPLC-FL. The experiments were performed with PY79, PY79 Δ*spsA* (Δ*spsA*), and PY79 Δ*spsB* (Δ*spsB*) strains (A) or PY79, PY79 *cotE*::*erm* (*cotE*::*erm*), PY79 *cotX*::*erm* (*cotX*::*erm*), and PY79 *cotZ*::*erm* (*cotZ*::*erm*) strains (B). The results were standardized by the OD_600_ of the spore preparations. ns, not significant.; ****, *P ≤ *0.0001 for Δ versus PY79 by Welch’s *t* test. The error bars represent the SDs of the means.

The final acceptor(s) of the Leg on the forespore surface as well as the enzyme(s) involved in the transfer of the Leg to the final acceptor(s) remained to be identified. The *spsA* and *spsB* genes encode putative glycosyltransferases (Table S1). Glycosyltransferases represent a subclass of enzymes that catalyze glycoside linkage synthesis by the transfer of a monosaccharide from an activated donor substrate to an acceptor. Therefore, the SpsA and SpsB proteins were candidates for transferring the Leg from the CMP-Leg to one or more acceptor(s) on the forespore surface. To test this hypothesis, spores of the PY79 Δ*spsA* and PY79 Δ*spsB* mutant strains were produced. Spores of both mutant strains were less hydrophilic and negatively charged than spores of the PY79 strain, and complementations restored the hydrophilic properties of spores of the PY79 strain (Fig. S5C, D, and E). This indicates that the *spsA* and *spsB* genes are involved in spore surface maturation. To determine whether the modifications of the surface properties of spores of the Δ*spsA* and Δ*spsB* mutant strains were due to a defect in Leg transfer to the forespore surface, the surface fraction of both mutants was analyzed in RP-HPLC-FL (Fig. 6A). Surprisingly, the amounts of Leg in the surface fractions of the Δ*spsA* and Δ*spsB* mutant strains were similar to that of the PY79 stain, indicating that *spsA* and *spsB* genes are not required for Leg transfer to the forespore surface.

### Legionaminic acid is linked to the crust.

To determine whether the Leg is linked to the crust or the outer coat, the spore surface fractions of the PY79 *cotE*::*erm*, PY79 *cotX*::*erm*, and PY79 *cotZ*::*erm* mutant strains were analyzed in RP-HPLC-FL (Fig. 6B). It was shown previously that spores of the *cotE* mutant strain lack both the outer coat and the crust, while spores of the *cotZ* mutant strains only lack the crust (14, 45). The *cotX* mutant stain has an assembled crust, but it is loosely attached to spores (14). However, in our hands, we observed that the hydrophilicity of spores of the *cotX* mutant strain decreased during the washing of the spore preparations, which would indicate that the crust is lost under our experimental conditions (data not shown). Therefore, if the Leg was linked to the crust, a decrease of the Leg amount was expected in the surface fractions of the *cotE*, *cotX*, and *cotZ* mutant strains by comparison with that of the PY79 strain. In contrast, if the Leg was linked to the outer coat, a decrease of the Leg amount was only expected in the surface fraction of the *cotE* mutant strain. The inactivation of the *cotE*, *cotX*, and *cotZ* genes strongly reduced the amount of Leg in the surface fraction of spores, indicating that Leg is linked to the crust.

## DISCUSSION

We previously showed in B. subtilis that the glycans of the crust are composed of rhamnose, quinovose, glucosamine and muramic lactam, this last being known to be part of the bacterial peptidoglycan (9). In this study, we showed that the surface of B. subtilis spores also contained Leg, a NulO identified for the first time on the surface of bacterial spores. NulOs are composed of nine-carbon-backbone monosaccharides differing by structural variations at the C-5, C-7, and C-9 positions (46). NulOs notably encompass sialic acids, such as Neu5Ac and the ketodeoxynonulosonic acid, as well as nonsialic acids, such as Leg and Pse. Biosynthesis of NulOs proceeds through a conserved enzymatic mechanism starting by the condensation of a 6-carbon monosaccharide with a 3-carbon pyruvate. The 9-carbon intermediate is then activated to a CMP-sugar, before being transferred to an acceptor. The CMP-Pse biosynthesis pathway of H. pylori is an example of a NulO pathway that has been extensively studied (31, 47, 48). In H. pylori, the nucleotide activated Pse is synthesized from UDP-GlcNAc by six consecutive enzymes. The CMP-Leg pathway in C. jejuni is quite similar, except that the sugar precursors are GDP linked, rather than UDP linked. It also differs in the epimerization performed at C-2, C-4, and C-5 of the 6-deoxy-hexoses intermediates, resulting in stereochemical differences at C-5, C-7, and C-8 of the final nonulosonates (30). In contrast to that of H. pylori, the Pse pathway of B. thuringiensis involves seven enzymes (43). The two pathways differ by the enzymes catalyzing the first reaction of the pathway, i.e., the synthesis of the UDP-4-keto-6-deoxy-AltNAc from the UDP-GlcNAc. In H. pylori this reaction is catalyzed by PseB, while it is catalyzed by Pen and Pal in B. thuringiensis. Interestingly, it was shown that the coincubation of Pen and Pal with UDP-GlcNAc *in vitro* leads to the formation of UDP-4-keto-6-deoxy-AltNAc and UDP-4-keto-6-deoxy-GlcNAc (43).

By using gene annotation and RaptorX software, we identified SpsM as the putative enzyme catalyzing the first reaction of the CMP-Leg pathway in B. subtilis, and we demonstrated that the *spsM* gene is required for Leg biosynthesis. We also showed that the insertion of the *pen-pal* and *pglF* genes into the *amyE* locus of the Δ*spsM* mutant strain restored Leg production. Pen-Pal and PglF were previously shown to convert UDP-GlcNAc to UDP-4-keto-6-deoxy-GlcNAc (43, 49). More surprisingly, the addition of the *legB* gene in the Δ*spsM* mutant strain did not fully restore Leg production. LegB is the first enzyme of the CMP-Leg pathway in C. jejuni, and it catalyzes the C-4/C-6 dehydration of the GDP-GlcNAc (30). These data indicate that SpsM is a C-4/C-6 dehydratase, but unlike LegB, it preferentially uses UDP-GlcNAc as a substrate rather than GDP-GlcNAc. These results also imply that the following enzymes of the CMP-Leg pathway in B. subtilis use UDP-linked precursors, indicating that the CMP-Leg pathway of B. subtilis differs from the only complete CMP-Leg pathway described to date in C. jejuni (30). By a similar approach to that carried out with SpsM, we identified SpsC, SpsD, SpsG, SpsE and SpsF as the following enzymes of the pathway, we predicted their functions (see Text S2 and Fig. S6 in the supplemental material), and we reconstructed the CMP-Leg pathway of B. subtilis (Fig. 3A). These *in silico* data are consistent with our experimental results, which showed that the *spsC*, *spsD*, *spsG*, *spsE* and *spsF* genes are required for CMP-Leg biosynthesis. To summarize, this study identified an original CMP-Leg pathway in B. subtilis consisting of six enzymes and using UDP-linked sugars as precursors.

10.1128/mBio.01153-20.6 FIG S6 Structure or predicted structure of the Sps proteins. (A) Cartoon representation of the tertiary structure prediction of SpsC. (Top) Tertiary structure prediction. (Bottom) Putative pyridoxal-5′-phosphate pocket. (B) Predicted secondary and tertiary structures of SpsD. (Top) Secondary structure prediction. (Bottom) Cartoon representation of the tertiary structure prediction. Two β-strands form a V-like shape named “β-bulge” that could constitute a binding site for acetyl coenzyme A (acetyl-CoA). (C) Cartoon representation of the tertiary structure prediction of SpsG. (Top) Tertiary structure prediction. (Bottom) Putative UDP binding pocket. (D) Cartoon representation of the tertiary structure of SpsE. Protein crystal structure was obtained by X-ray diffraction at a resolution of 2.38 Å by the Joint Center for Structural Genomics and was deposited in the PDB database under the reference 1VLI. (Top) Tertiary structure. (Middle) Focus on the 8 β-strands of the triosephosphate isomerase (TIM)-barrel fold. The 8 β-strands are highlighted in magenta. (Bottom) Putative PLP binding pocket. (E) Cartoon representation of the tertiary structure prediction of SpsF. (Top) Tertiary structure prediction. The SpsF monomer has an αβα three-layer sandwich architecture. (Middle) Focus on the seven β-strands with the topological order ↑β3-↑β2-↑β1-↑β4-↓β8-↑β5-↑β9 (from bottom to top). (Bottom) Putative CTP binding pocket. The tertiary structures and binding sites predictions were obtained with RaptorX software. The tertiary structure representations were performed with Chimera and Jmol software. α-Helix, β-strand, and coil are represented in cyan, magenta, and brown, respectively. Download FIG S6, PDF file, 1.5 MB. Copyright © 2020 Dubois et al. 2020 Dubois et al. This content is distributed under the terms of the Creative Commons Attribution 4.0 International license.

10.1128/mBio.01153-20.8 TEXT S2 Comment on the results of the structural analysis. Download Text S2, DOCX file, 0.1 MB. Copyright © 2020 Dubois et al. 2020 Dubois et al. This content is distributed under the terms of the Creative Commons Attribution 4.0 International license.

Once activated, the Leg must be transferred to the forespore surface by a specific transferase. There are currently no reports on the bacterial enzymes transferring Leg to cell surfaces. However, crystal structures of four bacterial sialyltransferases have been reported (50–54). These bacterial sialyltransferases have been classified into four carbohydrate-active enzyme database (CAZy) families, including GT38, GT42, GT52, and GT80, based on their amino acid sequence similarities (55, 56). However, in B. subtilis, no glycosyltransferase belongs to any of these four CAZy families. Due to their predicted glycosyltransferase function and the vicinity of the *spsA* and *spsB* genes to the genes of the CMP-Leg pathway, the SpsA and SpsB proteins were the most likely transferases of the CMP-Leg in B. subtilis. An analysis performed with the NCBI conserved domain database identified a putative TagB domain in the primary sequence of SpsB. The TagB protein is involved in the priming step of polyglycerol phosphate wall teichoic acid synthesis in B. subtilis (57). Interestingly, two enzymes containing a TagB domain have recently been identified as plausible candidates for a Pse and a Leg derivative transferase in the bacteria Tannerella forsythia (58). In addition, the protein sequence of SpsB contains one DEG and three HP domains. Both D/E-D/E-G and HP motifs are well conserved among different sialyltransferase families with otherwise little or no sequence identity, and these motifs were shown to be important for enzyme catalysis and CMP-Neu5Ac binding (59). Surprisingly, we showed that SpsB is not required for Leg transfer to the forespore surface. Nevertheless, at this stage, it is not possible to exclude that SpsB transfers another still unidentified NulO or that *spsB* deletion is compensated by another Leg transferase with another acceptor on the forespore surface. The SpsA protein is a transferase of the GT-2 glycosyltransferase family whose three-dimensional structure has been resolved in complex with Mn-dTDP and Mg-DTP (60, 61). The protein sequence of SpsA contains an asparagine doublet, acting as the DxD motif frequently found in the active site of glycosyltransferases. The asparagine interacts both with the ribose moiety of the nucleotide sugar donor and the divalent cation, thus stabilizing the charged phosphate groups in the nucleotide sugar donor (61). Sialyltransferases usually lack a DxD motif and do not require divalent metal cations for enzymatic activity (62). Accordingly, we showed that SpsA is not required for the transfer of Leg to the forespore surface. These findings suggest that SpsA is not a CMP-Leg transferase. It is more likely that SpsA participates in the transfer of a monosaccharide to a glycan which is being synthesized.

In this study, we showed that the Leg on the spore surface is linked to the crust. Furthermore, we demonstrated that the abolition of Leg production leads to a crust assembly defect. It was shown elsewhere that Pse is essential for flagellar assembly in *Campylobacter* spp. and H. pylori (32, 34). In H. pylori, single Pse moieties are attached by *O*-linkages to the structural flagellar proteins FlaA and FlaB, at up to 7 and 10 Ser/Thr residues, respectively. In C. jejuni, the flagellum is also decorated, but with more complex pseudaminic acids, including one with acetamido groups (34, 35, 63). More recently, it was also shown that Leg is present on the flagellins of C. jejuni NCTC 11168 and that flagellin glycosylation is highly heterogeneous, with up to 6 different sugars singly present at a given site (64). Therefore, it is likely that several proteins of the crust of B. subtilis spores are *O*-glycosylated with Leg on one or several glycosylation sites, and we hypothesize that these *O*-linked Leg participate in the assembly or the stabilization of the interactions between the crust proteins and/or in the anchoring of the crust to the outer coat. The way by which Leg stabilizes the interactions between the spore surface proteins remains to be defined. However, NulOs are well known to participate in carbohydrate-protein interactions, e.g., mediation of cell-cell adhesion via lectins or cell-cell communication (63). NulOs are hydrophilic and negatively charged at neutral pH and they typically occur as the terminating units of *N*-glycans, *O*-glycans, and glycolipids, which could explain the multiplicity of biological functions they fulfill. In this study, we showed that the abolition of Leg production resulted in a decrease in the negative charge and hydrophilicity of B. subtilis spores, making them more adherent to stainless steel. However, similar surface modifications were obtained with spores of the Δ*spsA* and Δ*spsB* mutant strains that still produce Leg, suggesting that the hydrophilicity and the negative charge of spores are provided by a crust glycan, rather than by Leg.

**FIG 7:**
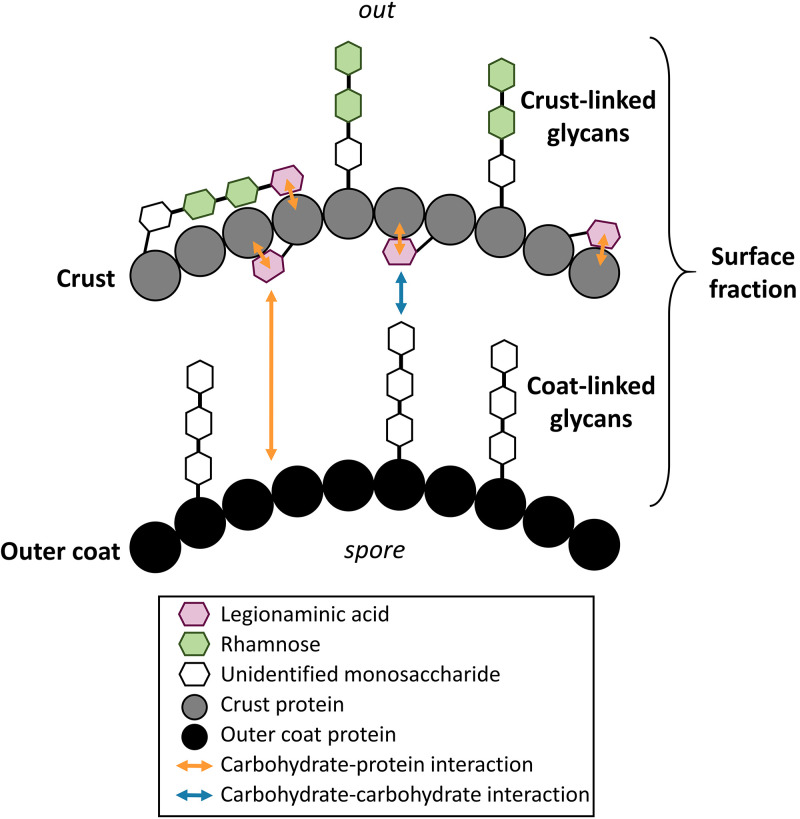
Hypothetical model of the B. subtilis spore surface organization. In this model, one or several crust proteins are glycosylated by Leg or by a crust-linked glycan containing Leg. Leg could participate in the assembly and/or the stabilization of the interactions between the crust proteins possibly through a carbohydrate-protein interaction. The crust proteins and the crust-linked glycan give the spores their hydrophilicity and negative charge that define the adhesion properties of B. subtilis spores. Leg might also participate in the anchoring of the crust proteins to the outer coat by interacting through a carbohydrate-protein interaction with the outer coat proteins or by a carbohydrate-carbohydrate interaction with a coat-linked glycan. The interactions presented in this model are speculative. They are extrapolated from current knowledge about NulOs in bacteria (see Discussion). In the absence of data about the structure of the coat-linked and crust-linked glycans, the number of monosaccharides that make up the glycans and the position of the bonds between monosaccharides are just indicative of a possible structure.

The total amounts of neutral sugars in the surface fractions of the Δ*spsABCDEF* and Δ*spsM* mutant strains were halved compared to that of the wild-type strain. Given that most of the crust is lacking on the spore surfaces of both mutants, this result suggests that approximately one-half of the monosaccharides are located on the outer coat surface and the other half are linked to the crust, which agrees with previous studies (15, 17). The precise localization, structure, and composition of the glycans containing these monosaccharides remain to be defined. However, it has been suggested that one of these glycans contains rhamnose and another one contains galactose (15). It was shown previously that *spsM* deletion leads to a loss of rhamnose on the spore surface, and we confirmed this by gas chromatography-mass spectrometry (GC-MS) experiments (data not shown) (16). Knowing the activity of SpsM, the loss of rhamnose in the Δ*spsM* mutant strain is obviously an indirect consequence of Leg production abolition. Since Leg is required for crust assembly, it is therefore likely that rhamnose moieties are constitutive of a crust-linked glycan. Using the data from this study, we propose a speculative model for the organization of the B. subtilis spore surface (Fig. 7).

In most of the B. subtilis strains, the *spsM* gene is interrupted by the spβ prophage. The prophage is excised from the chromosome both during sporulation and in response to stress (DNA damage), thus reconstituting a functional *spsM* gene (16). Another study indicates that the SigY sigma factor promotes the maintenance of spβ in the chromosome of B. subtilis (65). As the other ECF-type sigma factors, SigY activity is regulated by stresses and nitrogen deficiency has been shown to increase SigY activity (66). Therefore, it is likely that stresses encountered by B. subtilis during sporulation modulate the excision of spβ and consequently the production of Leg. Knowing that Leg is required for crust formation and that presence or absence of crust determines the surface and adhesion properties of spores, it is likely that the environmental conditions encountered by B. subtilis during sporulation influence the adhesion properties of spores. In the context of food industries, this could mean that production, cleaning and disinfection processes encountered by the sporulating *Bacillus* cells contaminating the production lines might influence the adhesion properties of the resulting spores. This adaptive strategy would explain the high persistence of these microorganisms on food industry production lines.

## MATERIALS AND METHODS

### Bacterial strains and growth conditions.

The strains used in this study are described in Table 1. Escherichia coli K-12 strain TG1 was used as host for the construction of plasmids and cloning experiments. B. subtilis cells were transformed as described previously (67). E. coli strains were grown at 37°C in Luria Broth (LB). *Bacillus* strains were grown at 30°C or 37°C in LB, Nutrient Broth (NB), or Spo8, a sporulation medium (68). The following concentrations of antibiotic were used for bacterial selection: 100 μg/ml ampicillin and 20 μg/ml chloramphenicol for E. coli, and 1 μg/ml erythromycin and 5 μg/ml chloramphenicol for *Bacillus*. Spores were produced at 30°C on Spo8-agar as previously described (68). After a 10-day incubation period and when over 95% of spores were obtained, spores were harvested by scraping the surfaces of plates, washing them five times in chilled sterile water (1,500 × *g* for 15 min), and storing them in sterile water at 4°C until use. The titer of spore preparations was evaluated by plating on NB-agar and by measuring the optical density at 600 nm (OD_600_).

**TABLE 1:** Strains used in this study

Strain	Trait or relevant genotype	Reference or source
*B. subtilis* PY79		BGSC, 1A747
*B. subtilis* PY79 Δ*spsABCDEF*	*spsABCDEF*-deficient strain	This study
*B. subtilis* PY79 Δ*spsA*	*spsA*-deficient strain; markerless	This study
*B. subtilis* PY79 Δ*spsB*	*spsB*-deficient strain; markerless	This study
*B. subtilis* PY79 Δ*spsD*	*spsD*-deficient strain; markerless	This study
*B. subtilis* PY79 Δ*spsF*	*spsF*-deficient strain; markerless	This study
*B. subtilis* PY79 Δs*psM*	*spsM*-deficient strain; markerless	This study
*B. subtilis* PY79 *cotE*::*erm*	*cotE*-inactivated strain; Ery^r^	This study
*B. subtilis* PY79 c*otX*::*erm*	*cotX*-inactivated strain; Ery^r^	This study
*B. subtilis* PY79 *cotZ*::*erm*	*cotZ*-inactivated strain; Ery^r^	This study
*B. subtilis* PY79 Δ*spsA amyE*::*spsA*	Complementation of the *spsA*-deficient strain; Cm^r^	This study
*B. subtilis* PY79 Δ*spsB amyE*::*spsB*	Complementation of the *spsB*-deficient strain; Cm^r^	This study
*B. subtilis* PY79 Δ*spsD amyE*::*spsD*	Complementation of the *spsD*-deficient strain; Cm^r^	This study
*B. subtilis* PY79 Δ*spsF amyE*::*spsF*	Complementation of the *spsF*-deficient strain; Cm^r^	This study
*B. subtilis* PY79 Δ*spsM amyE*::*spsM*	Complementation of the *spsM*-deficient strain; Cm^r^	This study
*B. subtilis* PY79 Δ*spsM amyE*::*spsM* M146A	M146A mutated allele of *spsM* in the amyE locus of the *spsM*-deficient strain; Cm^r^	This study
*B. subtilis* PY79 Δ*spsM amyE*::*spsM* K150A	K150A mutated allele of *spsM* in the *amyE* locus of the *spsM*-deficient strain; Cm^r^	This study
*B. subtilis* PY79 Δ*spsM amyE:*:*pen*	*pen* gene in the *amyE* locus of the *spsM*-deficient strain; Cm^r^	This study
*B. subtilis* PY79 Δ*spsM amyE*::*pal*	*pal* gene in the *amyE* locus of the *spsM*-deficient strain; Cm^r^	This study
*B. subtilis* PY79 Δ*spsM amyE*::*pen-pal*	*pen* and *pal* genes in the *amyE* locus of the *spsM*-deficient strain; Cm^r^	This study
*B. subtilis* PY79 Δ*spsM amyE*::*legB*	*legB* gene in the *amyE* locus of the *spsM*-deficient strain; Cm^r^	This study
*B. subtilis* PY79 Δ*spsM amyE*::*pglF*	*pglF* gene in the *amyE* locus of the *spsM*-deficient strain; Cm^r^	This study
*B. subtilis* PY79 Δ*spsM amyE*::*pseB*	*pseB* gene in the *amyE* locus of the *spsM*-deficient strain; Cm^r^	This study
B. subtilis PY79 *amyE*::P_spsA_-*mCherry*	P_spsA_-*mCherry* transcriptional fusion in the *amyE* locus of the PY79 strain; Cm^r^	This study
B. subtilis 168		BGSC, 1A1
B. subtilis 168 Δ*spsC*	*spsC*-deficient strain; Km^r^	BGSC, BKK37890
B. subtilis 168 Δ*spsE*	*spsE*-deficient strain; Km^r^	BGSC, BKK37870
B. subtilis 168 Δ*spsG*	*spsG*-deficient strain; Km^r^	BGSC, BKK37850
B. thuringiensis serovar *israelensis* ATCC 35646		BGSC, 4Q12

### DNA manipulations.

Total DNA was extracted from B. subtilis cells using the Puregene Yeast/Bact kit (Qiagen, France). Plasmid DNA was extracted from E. coli using a SmartPure plasmid kit (Eurogentec, Belgium). Restriction enzymes and T4 DNA ligase (New England BioLabs, USA) were used in accordance with the manufacturer's recommendations. Oligonucleotide primers were synthesized by Eurofins Genomics (Germany). PCRs were performed in a SimpliAmp thermal cycler (Applied Biosystems, USA). Amplified fragments were purified using the SmartPure PCR kit (Eurogentec, Belgium). Digested DNA fragments were separated on 1% (w/v) agarose gels after digestion and extracted from gels using the SmartPure gel kit (Eurogentec, Belgium). Nucleotide sequences of plasmid inserts were determined by Sanger sequencing (Eurofins Genomics, Germany).

### Construction of the B. subtilis recombinant strains.

Gene deletion and gene interruption were performed by homologous recombination with pMAD and pMUTIN4, respectively, as previously described (69, 70). The deletion mutants were constructed by deleting the targeted genes from the ATG to the stop codon without introducing exogenous DNA to avoid disrupting downstream genes transcription. Complementation was introduced in the *amyE* gene using the plasmid pBS1C (67). The recombinant strains, oligonucleotides, and plasmids used in this study are described in Table 1 and Tables S2A and B in the supplemental material. Nucleotide sequences of the introduced chromosomal modifications were verified by Sanger sequencing (Eurofins Genomics, Germany).

10.1128/mBio.01153-20.10 TABLE S2 Oligonucleotides and plasmids. (A) Oligonucleotides used in this study (B) Plasmids constructed for this study. Download Table S2, DOCX file, 0.1 MB. Copyright © 2020 Dubois et al. 2020 Dubois et al. This content is distributed under the terms of the Creative Commons Attribution 4.0 International license.

### Surface and adhesion properties of spores.

Microbial-adhesion-to-hydrocarbons (MATH), zetametry, and adhesion assays were performed as described previously (9).

### Electron microscopy.

To observe spore surface features, spores were fixed and stained as previously described (9). Ultrathin sections were cut on a Leica UC7 ultramicrotome and collected on 150-mesh copper grids. The sections were examined under a Jeol JEM-2100 transmission electron microscope at an accelerated voltage of 200 kV.

### Preparation of the intracellular extracts and spore surface fractions.

A cell pellet obtained by centrifugation (12,000 × *g*, 15 min, 4°C) and washed twice with cold sterile ultrapure water was resuspended in 3 ml of sodium citrate buffer (0.01 M, pH 5.5) and then transferred to a screw tube containing 400 mg of glass beads (acid-washed, G1277-500G; Sigma). The screw tube was shaken in a MiniBeadBeater-16 (Biospec Products) for 2 min and then cooled on ice. This treatment was repeated 4 times. The lysate was then centrifuged (6,000 × *g*, 15 min, 4°C), and 1 ml of the supernatant was ethanol precipitated. The precipitate was recovered in 500 μl of sterile ultrapure water and kept at −20°C for further analyses. The surface fraction of spores was removed by three successive passages of spore suspensions through a French press (SLM Instruments, Urbana, IL) at 20,000 lb/in^2^. Spores were separated from the surface fraction by two successive centrifugations (4,500 × *g*, 15 min, 4°C), and the supernatant was kept at −20°C until use.

### Dosage of neutral sugars.

The dosage of the neutral sugars in the surface fraction of spores was performed as described previously (28). The amounts of neutral sugars were standardized by the OD_600_ of the spore preparations.

### Release and derivation of NulOs.

500 μl of spore surface fraction or intracellular extract was lyophilized. DMB-coupled NulOs were obtained as described previously (36). Shortly, dried glycoconjugates were hydrolyzed at 80°C for 2 h in 0.1 M trifluoroacetic acid to release the NulOs. NulOs were subsequently coupled to DMB by heating the samples at 50°C for 2 h in the dark in 7 mM DMB, 1 M β-mercaptoethanol, 18 mM sodium hydrosulfite in 0.02 mM trifluoroacetic acid.

### Analysis of DMB derivatives on HPLC.

The NulO derivatives were separated isocratically on a C_18_ reverse-phase HPLC column (Waters, 4.6 mm by 150 mm, 3.5 μm. or Wakopak Handy ODS, 4.6 mm by 250 mm, 6 μm) using a solvent mixture of acetonitrile/methanol/water (7:9:84 [vol/vol/vol]), and they were detected by a fluorimeter (Waters 474, excitation wavelength [λ_exc_] = 373 nm, emission wavelength [λ_em_] = 448 nm). The Leg amount was estimated by measuring the area of peak 1 and by reporting this area to a standard range of Neu5Ac.

### Analysis of DMB derivatives on micro-LC/ESI-MS^3^.

Analyses were performed in positive ion mode on an amaZon speed ETD ion trap mass spectrometer equipped with the standard electrospray ionization (ESI) ion source and controlled by Hystar 3.2 software (Bruker Daltonics). DMB-coupled NulO separation was achieved on a micro-LC system (Prominence LC-20AB; Shimadzu, Kyoto, Japan). Samples were applied to a reverse-phase Luna C_18_-2 column (150 mm by 1.00 mm, 3-μm particles; Phenomenex), and they were separated using an isocratic elution of acetonitrile/methanol/water (6:4:90 [vol/vol/vol)] at a flow rate of 70 μl/min. The targeted MS^3^ scans for DMB-coupled NulO were performed using ultrascan mode (26,000 atomic mass units [amu]/s). NulO species were identified by referring to elution positions and MS^3^ fragmentation of Leg and Pse standards.

## References

[1] Gopal N, Hill C, Ross PR, Beresford TP, Fenelon MA, Cotter PD 2015 The prevalence and control of *Bacillus* and related spore-forming bacteria in the dairy industry. Front Microbiol 6:1418. doi:10.3389/fmicb.2015.01418. 26733963 PMC4685140

[2] Warth AD, Strominger JL 1969 Structure of the peptidoglycan of bacterial spores: occurrence of the lactam of muramic acid. Proc Natl Acad Sci U S A 64:528–535. doi:10.1073/pnas.64.2.528. 4982357 PMC223376

[3] Warth AD, Strominger JL 1972 Structure of the peptidoglycan from spores of *Bacillus subtilis*. Biochemistry 11:1389–1396. doi:10.1021/bi00758a010. 4259915

[4] Driks A, Eichenberger P 2016 The spore coat. Microbiol Spectr 4:TBS-0023-2016. doi:10.1128/microbiolspec.TBS-0023-2016. 27227299

[5] Driks A, Roels S, Beall B, Moran CP, Losick R 1994 Subcellular localization of proteins involved in the assembly of the spore coat of *Bacillus subtilis*. Genes Dev 8:234–244. doi:10.1101/gad.8.2.234. 8299942

[6] Ozin AJ, Henriques AO, Yi H, Moran CP 2000 Morphogenetic proteins SpoVID and SafA form a complex during assembly of the *Bacillus subtilis* spore coat. J Bacteriol 182:1828–1833. doi:10.1128/jb.182.7.1828-1833.2000. 10714986 PMC101864

[7] Roels S, Driks A, Losick R 1992 Characterization of spoIVA, a sporulation gene involved in coat morphogenesis in *Bacillus subtilis*. J Bacteriol 174:575–585. doi:10.1128/jb.174.2.575-585.1992. 1729246 PMC205752

[8] Takamatsu H, Kodama T, Nakayama T, Watabe K 1999 Characterization of the *yrbA* gene of *Bacillus subtilis*, involved in resistance and germination of spores. J Bacteriol 181:4986–4994. doi:10.1128/JB.181.16.4986-4994.1999. 10438771 PMC93988

[9] Faille C, Ronse A, Dewailly E, Slomianny C, Maes E, Krzewinski F, Guerardel Y 2014 Presence and function of a thick mucous layer rich in polysaccharides around *Bacillus subtilis* spores. Biofouling 30:845–858. doi:10.1080/08927014.2014.939073. 25115519

[10] Faille C, Cunault C, Dubois T, Bénézech T 2018 Hygienic design of food processing lines to mitigate the risk of bacterial food contamination with respect to environmental concerns. Innov Food Sci Emerg Technol 46:65–73. doi:10.1016/j.ifset.2017.10.002.

[11] Zhang J, Fitz-James PC, Aronson AI 1993 Cloning and characterization of a cluster of genes encoding polypeptides present in the insoluble fraction of the spore coat of *Bacillus subtilis*. J Bacteriol 175:3757–3766. doi:10.1128/jb.175.12.3757-3766.1993. 8509331 PMC204792

[12] McKenney PT, Driks A, Eskandarian HA, Grabowski P, Guberman J, Wang KH, Gitai Z, Eichenberger P 2010 A distance-weighted interaction map reveals a previously uncharacterized layer of the *Bacillus subtilis* spore coat. Curr Biol 20:934–938. doi:10.1016/j.cub.2010.03.060. 20451384 PMC2920530

[13] Imamura D, Kuwana R, Takamatsu H, Watabe K 2011 Proteins involved in formation of the outermost layer of *Bacillus subtilis* spores. J Bacteriol 193:4075–4080. doi:10.1128/JB.05310-11. 21665972 PMC3147665

[14] Shuster B, Khemmani M, Abe K, Huang X, Nakaya Y, Maryn N, Buttar S, Gonzalez AN, Driks A, Sato T, Eichenberger P 2019 Contributions of crust proteins to spore surface properties in *Bacillus subtilis*. Mol Microbiol 111:825–843. doi:10.1111/mmi.14194. 30582883 PMC6417949

[15] Bartels J, Blüher A, López Castellanos S, Richter M, Günther M, Mascher T 2019 The *Bacillus subtilis* endospore crust: protein interaction network, architecture and glycosylation state of a potential glycoprotein layer. Mol Microbiol 112:1576–1592. doi:10.1111/mmi.14381. 31502725

[16] Abe K, Kawano Y, Iwamoto K, Arai K, Maruyama Y, Eichenberger P, Sato T 2014 Developmentally-regulated excision of the SPβ prophage reconstitutes a gene required for spore envelope maturation in *Bacillus subtilis*. PLoS Genet 10:e1004636. doi:10.1371/journal.pgen.1004636. 25299644 PMC4191935

[17] Shuster B, Khemmani M, Nakaya Y, Holland G, Iwamoto K, Abe K, Imamura D, Maryn N, Driks A, Sato T, Eichenberger P 2019 Expansion of the spore surface polysaccharide layer in *Bacillus subtilis* by deletion of genes encoding glycosyltransferases and glucose modification enzymes. J Bacteriol 201:e00321-19. doi:10.1128/JB.00321-19. 31235516 PMC6755746

[18] Cangiano G, Sirec T, Panarella C, Isticato R, Baccigalupi L, De Felice M, Ricca E 2014 The *sps* gene products affect the germination, hydrophobicity, and protein adsorption of *Bacillus subtilis* spores. Appl Environ Microbiol 80:7293–7302. doi:10.1128/AEM.02893-14. 25239894 PMC4249184

[19] Plata G, Fuhrer T, Hsiao TL, Sauer U, Vitkup D 2012 Global probabilistic annotation of metabolic networks enables enzyme discovery. Nat Chem Biol 8:848–854. doi:10.1038/nchembio.1063. 22960854 PMC3696893

[20] Shornikov A, Tran H, Macias J, Halavaty AS, Minasov G, Anderson WF, Kuhn ML 2017 Structure of the *Bacillus anthracis* dTDP-l-rhamnose-biosynthetic enzyme dTDP-4-dehydrorhamnose 3,5-epimerase (RfbC). Acta Crystallogr F Struct Biol Commun 73:664–671. doi:10.1107/S2053230X17015849. 29199987 PMC5713671

[21] Gokey T, Halavaty AS, Minasov G, Anderson WF, Kuhn ML 2018 Structure of the *Bacillus anthracis* dTDP-l-rhamnose biosynthetic pathway enzyme: dTDP-α-d-glucose 4,6-dehydratase, RfbB. J Struct Biol 202:175–181. doi:10.1016/j.jsb.2018.01.006. 29331609 PMC5864537

[22] Law A, Stergioulis A, Halavaty AS, Minasov G, Anderson WF, Kuhn ML 2017 Structure of the *Bacillus anthracis* dTDP-l-rhamnose-biosynthetic enzyme dTDP-4-dehydrorhamnose reductase (RfbD). Acta Crystallogr F Struct Biol Commun 73:644–650. doi:10.1107/S2053230X17015746. 29199984 PMC5713668

[23] Baumgartner J, Lee J, Halavaty AS, Minasov G, Anderson WF, Kuhn ML 2017 Structure of the *Bacillus anthracis* dTDP-l-rhamnose-biosynthetic enzyme glucose-1-phosphate thymidylyltransferase (RfbA). Acta Crystallogr F Struct Biol Commun 73:621–628. doi:10.1107/S2053230X17015357. 29095156 PMC5683032

[24] Eichenberger P, Fujita M, Jensen ST, Conlon EM, Rudner DZ, Wang ST, Ferguson C, Haga K, Sato T, Liu JS, Losick R 2004 The program of gene transcription for a single differentiating cell type during sporulation in *Bacillus subtilis*. PLoS Biol 2:e328. doi:10.1371/journal.pbio.0020328. 15383836 PMC517825

[25] Steil L, Serrano M, Henriques AO, Völker U 2005 Genome-wide analysis of temporally regulated and compartment-specific gene expression in sporulating cells of *Bacillus subtilis*. Microbiology 151:399–420. doi:10.1099/mic.0.27493-0. 15699190

[26] Nicolas P, Mäder U, Dervyn E, Rochat T, Leduc A, Pigeonneau N, Bidnenko E, Marchadier E, Hoebeke M, Aymerich S, Becher D, Bisicchia P, Botella E, Delumeau O, Doherty G, Denham EL, Fogg MJ, Fromion V, Goelzer A, Hansen A, Härtig E, Harwood CR, Homuth G, Jarmer H, Jules M, Klipp E, Le Chat L, Lecointe F, Lewis P, Liebermeister W, March A, Mars RAT, Nannapaneni P, Noone D, Pohl S, Rinn B, Rügheimer F, Sappa PK, Samson F, Schaffer M, Schwikowski B, Steil L, Stülke J, Wiegert T, Devine KM, Wilkinson AJ, van Dijl JM, Hecker M, Völker U, Bessières P, Noirot P 2012 Condition-dependent transcriptome reveals high-level regulatory architecture in *Bacillus subtilis*. Science 335:1103–1106. doi:10.1126/science.1206848. 22383849

[27] Faille C, Lequette Y, Ronse A, Slomianny C, Garénaux E, Guerardel Y 2010 Morphology and physico-chemical properties of *Bacillus* spores surrounded or not with an exosporium: consequences on their ability to adhere to stainless steel. Int J Food Microbiol 143:125–135. doi:10.1016/j.ijfoodmicro.2010.07.038. 20739077

[28] Dubois M, Gilles K, Hamilton JK, Rebers PA, Smith F 1951 A colorimetric method for the determination of sugars. Nature 168:167. doi:10.1038/168167a0. 14875032

[29] Källberg M, Wang H, Wang S, Peng J, Wang Z, Lu H, Xu J 2012 Template-based protein structure modeling using the RaptorX web server. Nat Protoc 7:1511–1522. doi:10.1038/nprot.2012.085. 22814390 PMC4730388

[30] Schoenhofen IC, Vinogradov E, Whitfield DM, Brisson J-R, Logan SM 2009 The CMP-legionaminic acid pathway in *Campylobacter*: biosynthesis involving novel GDP-linked precursors. Glycobiology 19:715–725. doi:10.1093/glycob/cwp039. 19282391

[31] Schoenhofen IC, McNally DJ, Brisson J-R, Logan SM 2006 Elucidation of the CMP-pseudaminic acid pathway in *Helicobacter pylori*: synthesis from UDP-*N*-acetylglucosamine by a single enzymatic reaction. Glycobiology 16:8C–14C. doi:10.1093/glycob/cwl010. 16751642

[32] Goon S, Kelly JF, Logan SM, Ewing CP, Guerry P 2003 Pseudaminic acid, the major modification on *Campylobacter flagellin*, is synthesized via the Cj1293 gene. Mol Microbiol 50:659–671. doi:10.1046/j.1365-2958.2003.03725.x. 14617187

[33] Guerry P, Ewing CP, Schirm M, Lorenzo M, Kelly J, Pattarini D, Majam G, Thibault P, Logan S 2006 Changes in flagellin glycosylation affect *Campylobacter* autoagglutination and virulence. Mol Microbiol 60:299–311. doi:10.1111/j.1365-2958.2006.05100.x. 16573682 PMC1424674

[34] Schirm M, Soo EC, Aubry AJ, Austin J, Thibault P, Logan SM 2003 Structural, genetic and functional characterization of the flagellin glycosylation process in *Helicobacter pylori*. Mol Microbiol 48:1579–1592. doi:10.1046/j.1365-2958.2003.03527.x. 12791140

[35] Thibault P, Logan SM, Kelly JF, Brisson JR, Ewing CP, Trust TJ, Guerry P 2001 Identification of the carbohydrate moieties and glycosylation motifs in *Campylobacter jejuni* flagellin. J Biol Chem 276:34862–34870. doi:10.1074/jbc.M104529200. 11461915

[36] Sulzenbacher G, Roig-Zamboni V, Lebrun R, Guérardel Y, Murat D, Mansuelle P, Yamakawa N, Qian X-X, Vincentelli R, Bourne Y, Wu L-F, Alberto F 2018 Glycosylate and move! The glycosyltransferase Maf is involved in bacterial flagella formation. Environ Microbiol 20:228–240. doi:10.1111/1462-2920.13975. 29076618

[37] Lewis AL, Desa N, Hansen EE, Knirel YA, Gordon JI, Gagneux P, Nizet V, Varki A 2009 Innovations in host and microbial sialic acid biosynthesis revealed by phylogenomic prediction of nonulosonic acid structure. Proc Natl Acad Sci U S A 106:13552–13557. doi:10.1073/pnas.0902431106. 19666579 PMC2726416

[38] Ricaldi JN, Matthias MA, Vinetz JM, Lewis AL 2012 Expression of sialic acids and other nonulosonic acids in *Leptospira*. BMC Microbiol 12:161. doi:10.1186/1471-2180-12-161. 22853805 PMC3438082

[39] Miyafusa T, Caaveiro JMM, Tanaka Y, Tsumoto K 2013 Dynamic elements govern the catalytic activity of CapE, a capsular polysaccharide-synthesizing enzyme from *Staphylococcus aureus*. FEBS Lett 587:3824–3830. doi:10.1016/j.febslet.2013.10.009. 24157361

[40] Schoenhofen IC, McNally DJ, Vinogradov E, Whitfield D, Young NM, Dick S, Wakarchuk WW, Brisson J-R, Logan SM 2006 Functional characterization of dehydratase/aminotransferase pairs from *Helicobacter* and *Campylobacter*: enzymes distinguishing the pseudaminic acid and bacillosamine biosynthetic pathways. J Biol Chem 281:723–732. doi:10.1074/jbc.M511021200. 16286454

[41] Mulrooney EF, Poon KKH, McNally DJ, Brisson J-R, Lam JS 2005 Biosynthesis of UDP-*N*-acetyl-l-fucosamine, a precursor to the biosynthesis of lipopolysaccharide in *Pseudomonas aeruginosa* serotype O11. J Biol Chem 280:19535–19542. doi:10.1074/jbc.M500612200. 15778500

[42] Miyafusa T, Caaveiro JMM, Tanaka Y, Tanner ME, Tsumoto K 2013 Crystal structure of the capsular polysaccharide synthesizing protein CapE of *Staphylococcus aureus*. Biosci Rep 33:e00043. doi:10.1042/BSR20130017. 23611437 PMC3699295

[43] Li Z, Hwang S, Ericson J, Bowler K, Bar-Peled M 2015 Pen and Pal are nucleotide-sugar dehydratases that convert UDP-GlcNAc to UDP-6-deoxy-d-GlcNAc-5,6-ene and then to UDP-4-keto-6-deoxy-l-AltNAc for CMP-pseudaminic acid synthesis in *Bacillus thuringiensis*. J Biol Chem 290:691–704. doi:10.1074/jbc.M114.612747. 25414257 PMC4294493

[44] Koo B-M, Kritikos G, Farelli JD, Todor H, Tong K, Kimsey H, Wapinski I, Galardini M, Cabal A, Peters JM, Hachmann A-B, Rudner DZ, Allen KN, Typas A, Gross CA 2017 Construction and analysis of two genome-scale deletion libraries for *Bacillus subtilis*. Cell Syst 4:291.e7–305.e7. doi:10.1016/j.cels.2016.12.013. 28189581 PMC5400513

[45] Zheng LB, Donovan WP, Fitz-James PC, Losick R 1988 Gene encoding a morphogenic protein required in the assembly of the outer coat of the *Bacillus subtilis* endospore. Genes Dev 2:1047–1054. doi:10.1101/gad.2.8.1047. 3139490

[46] Varki A, Schnaar RL, Schauer R 2017 Sialic acids and other nonulosonic acids Essentials of Glycobiology. Cold Spring Harbor Laboratory Press, Cold Spring Harbor, NY.

[47] Ud-Din AI, Liu YC, Roujeinikova A 2015 Crystal structure of *Helicobacter pylori* pseudaminic acid biosynthesis *N*-acetyltransferase PseH: implications for substrate specificity and catalysis. PLoS One 10:e0115634. doi:10.1371/journal.pone.0115634. 25781966 PMC4363471

[48] Rangarajan ES, Proteau A, Cui Q, Logan SM, Potetinova Z, Whitfield D, Purisima EO, Cygler M, Matte A, Sulea T, Schoenhofen IC 2009 Structural and functional analysis of *Campylobacter jejuni* PseG: a UDP-sugar hydrolase from the pseudaminic acid biosynthetic pathway. J Biol Chem 284:20989–21000. doi:10.1074/jbc.M109.012351. 19483088 PMC2742864

[49] Riegert AS, Thoden JB, Schoenhofen IC, Watson DC, Young NM, Tipton PA, Holden HM 2017 Structural and biochemical investigation of PglF from *Campylobacter jejuni* reveals a new mechanism for a member of the short chain dehydrogenase/reductase superfamily. Biochemistry 56:6030–6040. doi:10.1021/acs.biochem.7b00910. 29053280 PMC6211297

[50] Chiu CPC, Watts AG, Lairson LL, Gilbert M, Lim D, Wakarchuk WW, Withers SG, Strynadka NCJ 2004 Structural analysis of the sialyltransferase CstII from *Campylobacter jejuni* in complex with a substrate analog. Nat Struct Mol Biol 11:163–170. doi:10.1038/nsmb720. 14730352

[51] Chiu CPC, Lairson LL, Gilbert M, Wakarchuk WW, Withers SG, Strynadka N 2007 Structural analysis of the α-2,3-sialyltransferase Cst-I from *Campylobacter jejuni* in apo and substrate-analogue bound forms. Biochemistry 46:7196–7204. doi:10.1021/bi602543d. 17518445

[52] Ni L, Chokhawala HA, Cao H, Henning R, Ng L, Huang S, Yu H, Chen X, Fisher AJ 2007 Crystal structures of *Pasteurella multocida* sialyltransferase complexes with acceptor and donor analogues reveal substrate binding sites and catalytic mechanism. Biochemistry 46:6288–6298. doi:10.1021/bi700346w. 17487984

[53] Ni L, Sun M, Yu H, Chokhawala H, Chen X, Fisher AJ 2006 Cytidine 5′-monophosphate (CMP)-induced structural changes in a multifunctional sialyltransferase from *Pasteurella multocida*. Biochemistry 45:2139–2148. doi:10.1021/bi0524013. 16475803

[54] Kakuta Y, Okino N, Kajiwara H, Ichikawa M, Takakura Y, Ito M, Yamamoto T 2008 Crystal structure of *Vibrionaceae Photobacterium* sp. JT-ISH-224 2,6-sialyltransferase in a ternary complex with donor product CMP and acceptor substrate lactose: catalytic mechanism and substrate recognition. Glycobiology 18:66–73. doi:10.1093/glycob/cwm119. 17962295

[55] Campbell JA, Davies GJ, Bulone V, Henrissat B 1997 A classification of nucleotide-diphospho-sugar glycosyltransferases based on amino acid sequence similarities. Biochemical J 326:929–939. doi:10.1042/bj3260929u. PMC1218753 9334165

[56] Coutinho PM, Deleury E, Davies GJ, Henrissat B 2003 An evolving hierarchical family classification for glycosyltransferases. J Mol Biol 328:307–317. doi:10.1016/S0022-2836(03)00307-3. 12691742

[57] Swoboda JG, Campbell J, Meredith TC, Walker S 2010 Wall teichoic acid function, biosynthesis, and inhibition. Chembiochem 11:35–45. doi:10.1002/cbic.200900557. 19899094 PMC2798926

[58] Tomek MB, Janesch B, Maresch D, Windwarder M, Altmann F, Messner P, Schäffer C 2017 A pseudaminic acid or a legionaminic acid derivative transferase is strain-specifically implicated in the general protein *O*-glycosylation system of the periodontal pathogen *Tannerella forsythia*. Glycobiology 27:555–567. doi:10.1093/glycob/cwx019. 28334934 PMC5420450

[59] Freiberger F, Claus H, Günzel A, Oltmann-Norden I, Vionnet J, Mühlenhoff M, Vogel U, Vann WF, Gerardy-Schahn R, Stummeyer K 2007 Biochemical characterization of a *Neisseria meningitidis* polysialyltransferase reveals novel functional motifs in bacterial sialyltransferases. Mol Microbiol 65:1258–1275. doi:10.1111/j.1365-2958.2007.05862.x. 17662040 PMC2169525

[60] Charnock SJ, Davies GJ 1999 Structure of the nucleotide-diphospho-sugar transferase, SpsA from *Bacillus subtilis*, in native and nucleotide-complexed forms. Biochemistry 38:6380–6385. doi:10.1021/bi990270y. 10350455

[61] Tarbouriech N, Charnock SJ, Davies GJ 2001 Three-dimensional structures of the Mn and Mg dTDP complexes of the family GT-2 glycosyltransferase SpsA: a comparison with related NDP-sugar glycosyltransferases. J Mol Biol 314:655–661. doi:10.1006/jmbi.2001.5159. 11733986

[62] Brockhausen I 2014 Crossroads between bacterial and mammalian glycosyltransferases. Front Immunol 5:492. doi:10.3389/fimmu.2014.00492. 25368613 PMC4202792

[63] Logan SM, Kelly JF, Thibault P, Ewing CP, Guerry P 2002 Structural heterogeneity of carbohydrate modifications affects serospecificity of *Campylobacter* flagellins. Mol Microbiol 46:587–597. doi:10.1046/j.1365-2958.2002.03185.x. 12406231

[64] Zebian N, Merkx-Jacques A, Pittock PP, Houle S, Dozois CM, Lajoie GA, Creuzenet C 2016 Comprehensive analysis of flagellin glycosylation in *Campylobacter jejuni* NCTC 11168 reveals incorporation of legionaminic acid and its importance for host colonization. Glycobiology 26:386–397. doi:10.1093/glycob/cwv104. 26582606

[65] Mendez R, Gutierrez A, Reyes J, Má R-, Magañ L 2012 The extracytoplasmic function sigma factor SigY is important for efficient maintenance of the Spb prophage that encodes sublancin in *Bacillus subtilis*. DNA Cell Biol 31:946–955. doi:10.1089/dna.2011.1513. 22400495 PMC3378957

[66] Tojo S, Matsunaga M, Matsumoto T, Kang C-M, Yamaguchi H, Asai K, Sadaie Y, Yoshida K-i, Fujita Y 2003 Organization and expression of the *Bacillus subtilis sigY* operon. J Biochem 134:935–946. doi:10.1093/jb/mvg225. 14769884

[67] Radeck J, Kraft K, Bartels J, Cikovic T, Dürr F, Emenegger J, Kelterborn S, Sauer C, Fritz G, Gebhard S, Mascher T 2013 The Bacillus BioBrick Box: generation and evaluation of essential genetic building blocks for standardized work with *Bacillus subtilis*. J Biol Eng 7:29. doi:10.1186/1754-1611-7-29. 24295448 PMC4177231

[68] Faille C, Bénézech T, Blel W, Ronse A, Ronse G, Clarisse M, Slomianny C 2013 Role of mechanical vs. chemical action in the removal of adherent *Bacillus* spores during CIP procedures. Food Microbiol 33:149–157. doi:10.1016/j.fm.2012.09.010. 23200646

[69] Vagner V, Dervyn E, Ehrlich D 1998 A vector for systematic gene inactivation in *Bacillus subtilis*. Microbiology 144:3097–3104. doi:10.1099/00221287-144-11-3097. 9846745

[70] Arnaud M, Chastanet A, Débarbouillé M 2004 New vector for efficient allelic replacement in naturally nontransformable, low-GC-content, Gram-positive bacteria. Appl Environ Microbiol 70:6887–6891. doi:10.1128/AEM.70.11.6887-6891.2004. 15528558 PMC525206

