# Supplementary material for: The sps Genes Encode an Original Legionaminic Acid Pathway Required for Crust Assembly in Bacillus subtilis
Source: mBio. 2020 Aug 18;11(4):e01153-20. doi: 10.1128/mBio.01153-20 (PMC7439481; doi:10.1128/mBio.01153-20)
Supplement: TABLE S1 [file mBio.01153-20-st001.docx]

|  |  |  | **RaptorX prediction** | | | | | |
| --- | --- | --- | --- | --- | --- | --- | --- | --- |
| Gene | putative function of the product | **Protein** | **P-value** | **Score** | **PDB Template** | **Organism** | **Protein** | **Activity** |
| *spsA* | dTDP-glycosyltransferase | **SpsA** | 8.64e-08 | 218 | 1H7L | *Bacillus subtilis* | SpsA | GT2 Glycosyltransferase |
| *spsB* | dTDP glycosyl/glycerophosphate transferase | **SpsB** | 6.41e-18 | 257 | 3MBO | *Bacillus anthracis* | BaBshA | Glycosyltransferase |
| *spsC* | Glutamine-dependent sugar transaminase | **SpsC** | 1.66e-09 | 348 | 1MDO | *Salmonella typhimurium* | ArnB | Aminotransferase |
| *spsD* | TDP-glycosamine N-acetyltransferase | **SpsD** | 7.32e-07 | 213 | 5KF1 | *Clostridium acetobutylicum* | GlmA | GNAT superfamily N-acetyltransferase |
| *spsE* | Phosphoenolpyruvate sugar pyruvyltransferase | **SpsE** | 3.20e-12 | 399 | 1VLI | *Bacillus subtilis* | SpsE | Unknown |
| *spsF* | glycosyltransferase | **SpsF** | 2.41e-07 | 192 | 6OEW | Leptospira borgpetersenii | - | cytidylyltransferase |
| *spsG* | glycosyltransferase | **SpsG** | 1.14e-06 | 253 | 3S2U | *Pseudomonas aeruginosa* | MurG | UDP-sugar hydrolase |
| *spsM* | dehydratase | **SpsM** | 4.17e-08 | 299 | 3VVC | *Staphylococcus aureus* | CapE | dehydrogenase/reductase |
|  |  |  | 8.81e-08 | 287 | 4J2O | *Acinetobacter baumannii* | WbjB | UDP-N-acetylglucosamine 4,6-dehydratase/5-epimerase |
|  |  |  | 2.00e-07 | 274 | 2GN4 | *Helicobacter pylori* | PseB | UDP-GlcNAc C6 dehydratase |
