# Supplementary figures and images for: The sps Genes Encode an Original Legionaminic Acid Pathway Required for Crust Assembly in Bacillus subtilis
Source: mBio. 2020 Aug 18;11(4):e01153-20. doi: 10.1128/mBio.01153-20 (PMC7439481; doi:10.1128/mBio.01153-20)

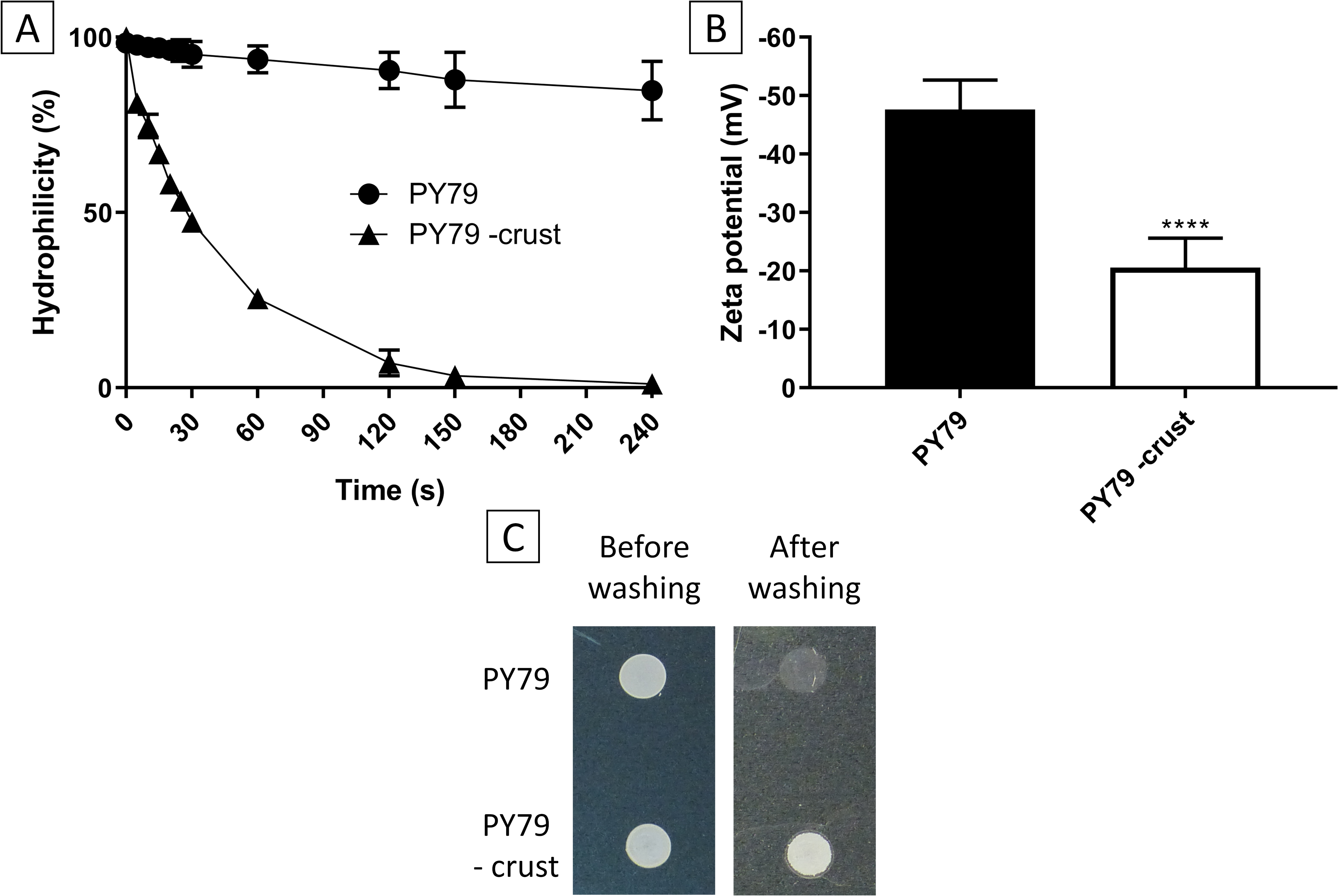

Supplement: FIG S1 [file mBio.01153-20-sf001.tif]

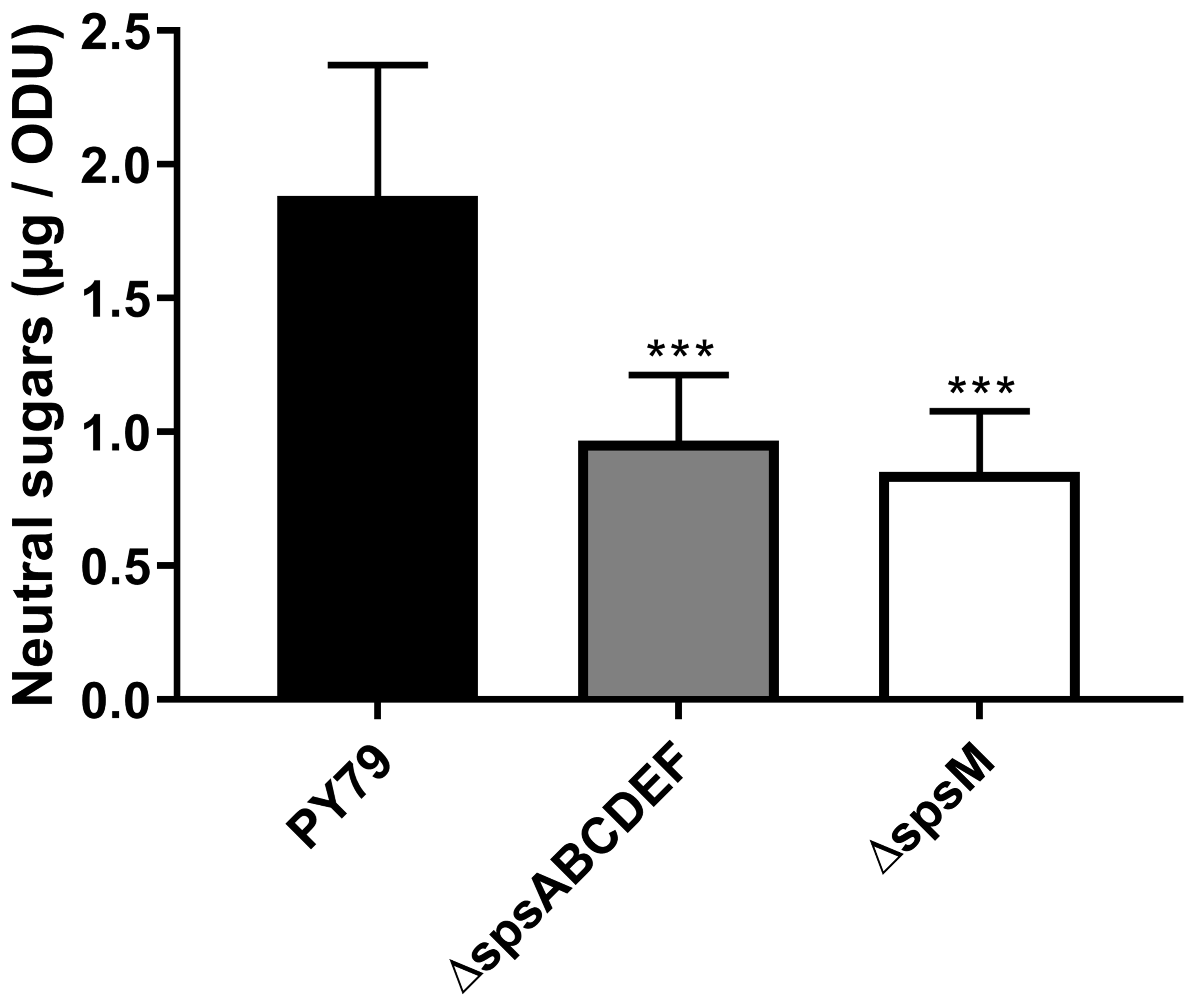

Supplement: FIG S2 [file mBio.01153-20-sf002.tif]

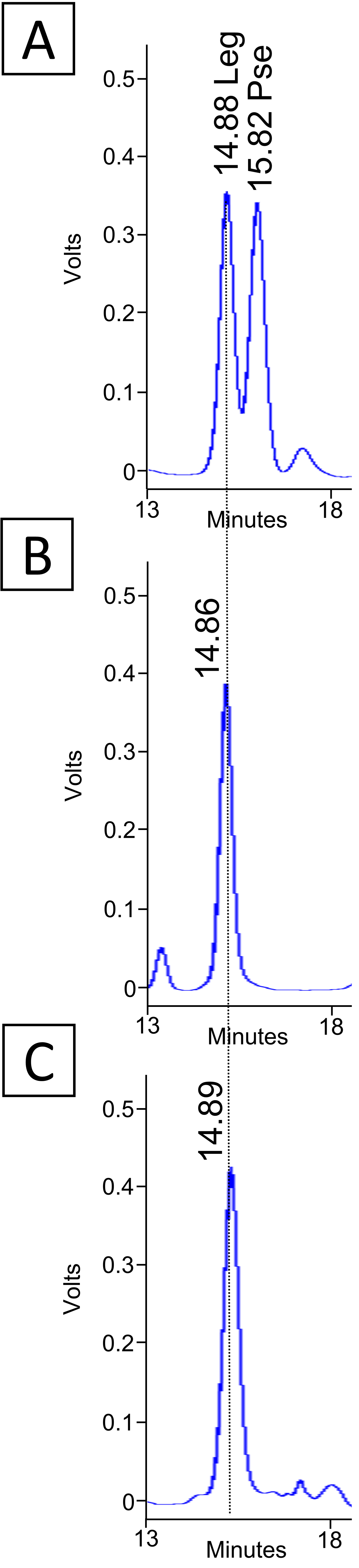

Supplement: FIG S3 [file mBio.01153-20-sf003.tif]

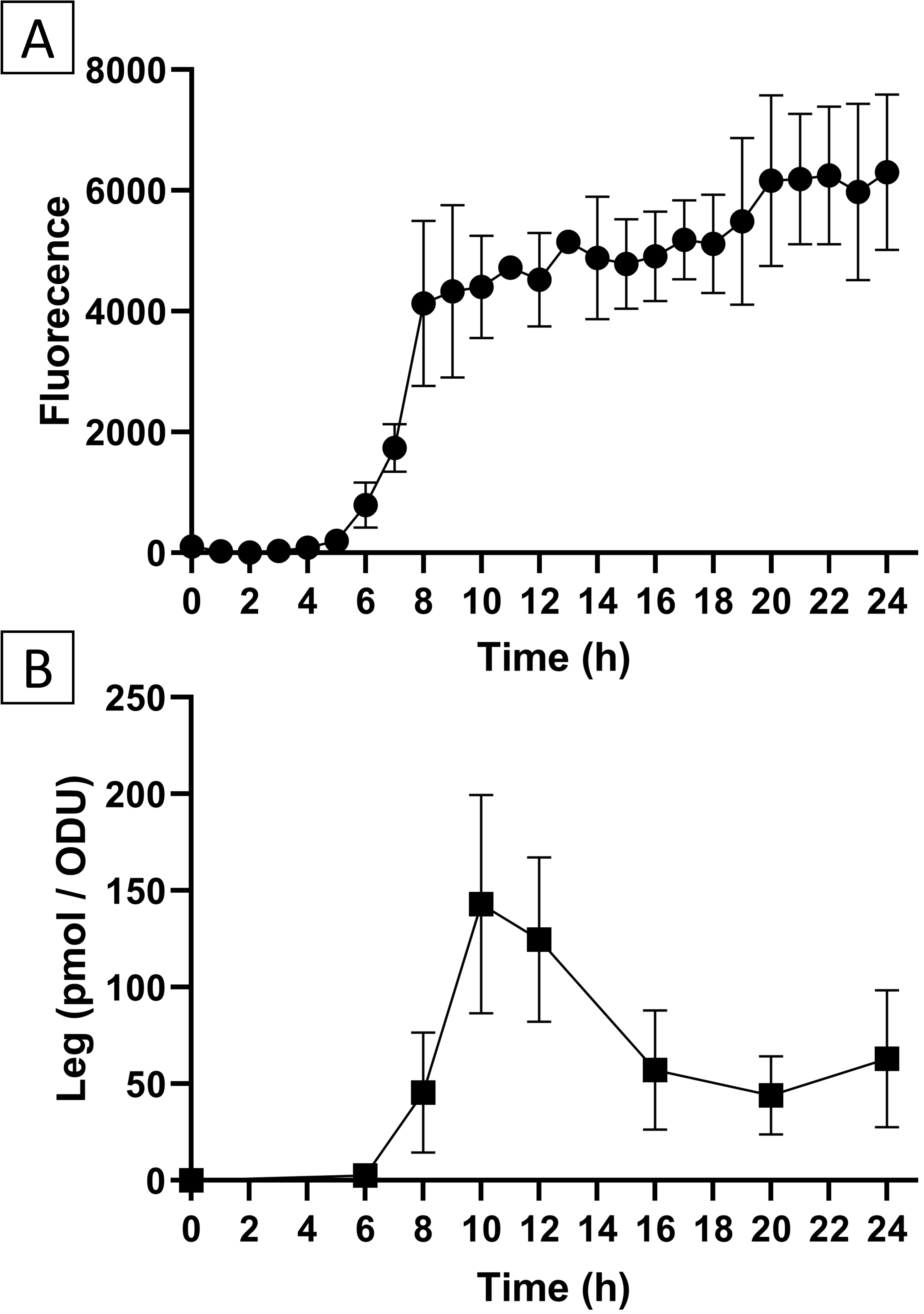

Supplement: FIG S4 [file mBio.01153-20-sf004.tif]

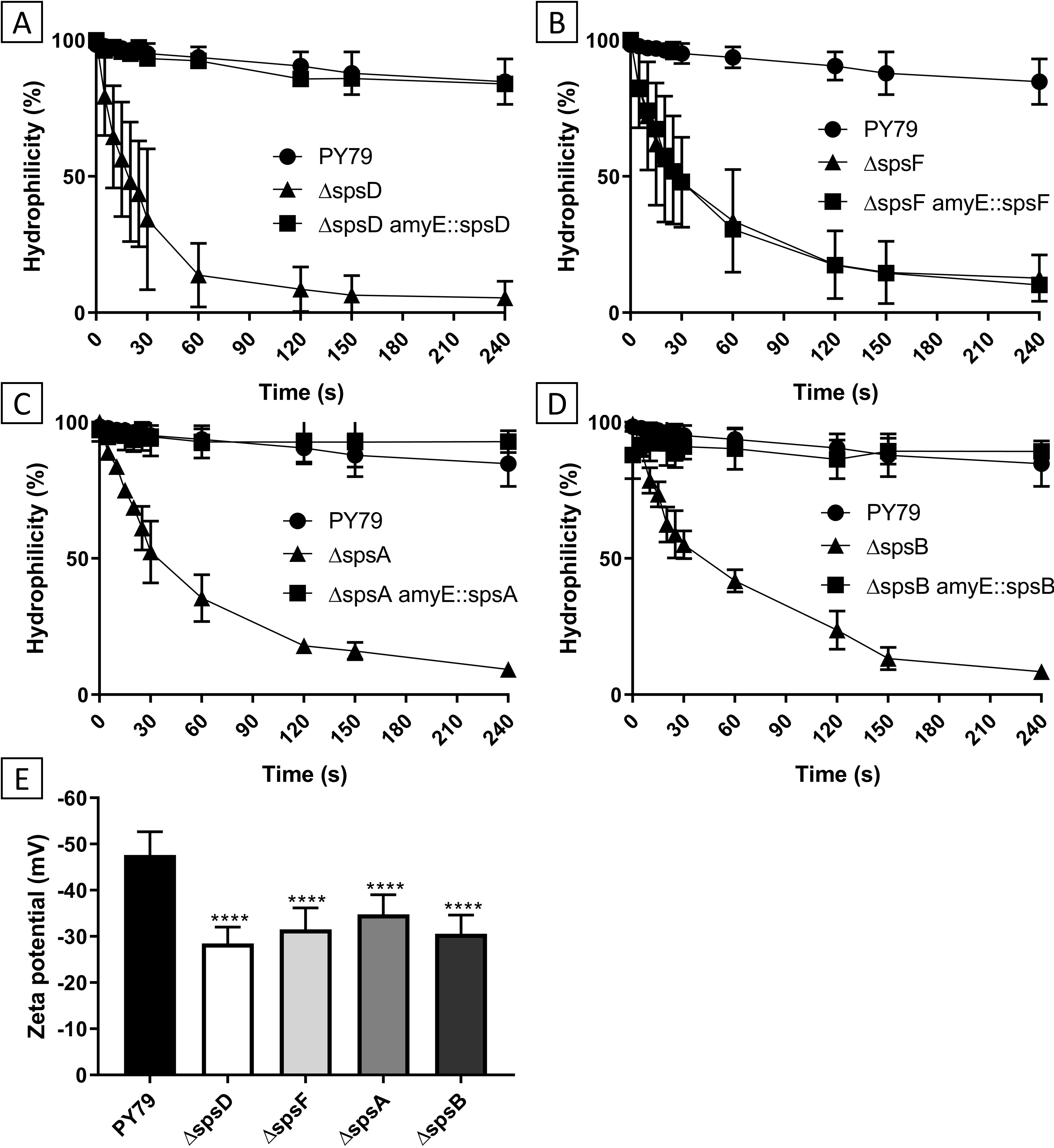

Supplement: FIG S5 [file mBio.01153-20-sf005.tif]
