# Supplementary material for: The sps Genes Encode an Original Legionaminic Acid Pathway Required for Crust Assembly in Bacillus subtilis
Source: mBio. 2020 Aug 18;11(4):e01153-20. doi: 10.1128/mBio.01153-20 (PMC7439481; doi:10.1128/mBio.01153-20)
Supplement: TEXT S1 [file mBio.01153-20-s0001.docx]

Despite several attemps, we failed to complement the ∆*spsF* mutant strain. However, with the data provided in this study, we believe that this lack of complementation does not question the conclusions about the role of *spsF* in CMP-Leg biosynthesis. To our knowledge, there are two reasons for complementing a mutant. The first is to verify that the introduced mutation does not disrupt the transcription of the flanking genes. The genes flanking the *spsF* gene are *spsE* (upstream) and *spsG* (downstream). We showed in this study that *spsF* and *spsE* genes are required for Leg biosynthesis (**Fig. 5**). Therefore, disruption of one of these genes in the ∆*spsF* mutant strain should abolished Leg production. The PY79 ∆*spsF* mutant strain still produced Leg (**Fig. 5B**), indicating that flanking genes transcription is not disturbed in the ∆*spsF* mutant strain. The second is to check that the phenotypes obtained with a mutant are not due to a mutation elsewhere on the chromosome. The ∆*spsF* mutant strain and the complemented strain were constructed twice and we obtained twice the same result (negative complementation, data not shown). In addition, structure and function predictions indicated that SpsF is a putative cytidylytransferase (**Text S2** and **Fig. S6**). Cytidylytransferases activate the NulOs and they are required for NulOs transfer to their acceptors. The phenotypes obtained with the ∆*spsF* mutant strain (Leg produced but not transferred to the forespore surface) perfectly match with the predicted cytidylytransferase activity of SpsF. Therefore, we are convinced of the veracity of the ∆*spsF* mutant strain phenotypes we reported in this study, despite the lack of complementation.
