# Supplementary material for: The sps Genes Encode an Original Legionaminic Acid Pathway Required for Crust Assembly in Bacillus subtilis
Source: mBio. 2020 Aug 18;11(4):e01153-20. doi: 10.1128/mBio.01153-20 (PMC7439481; doi:10.1128/mBio.01153-20)
Supplement: FIG S6 [file mBio.01153-20-sf006.pdf]

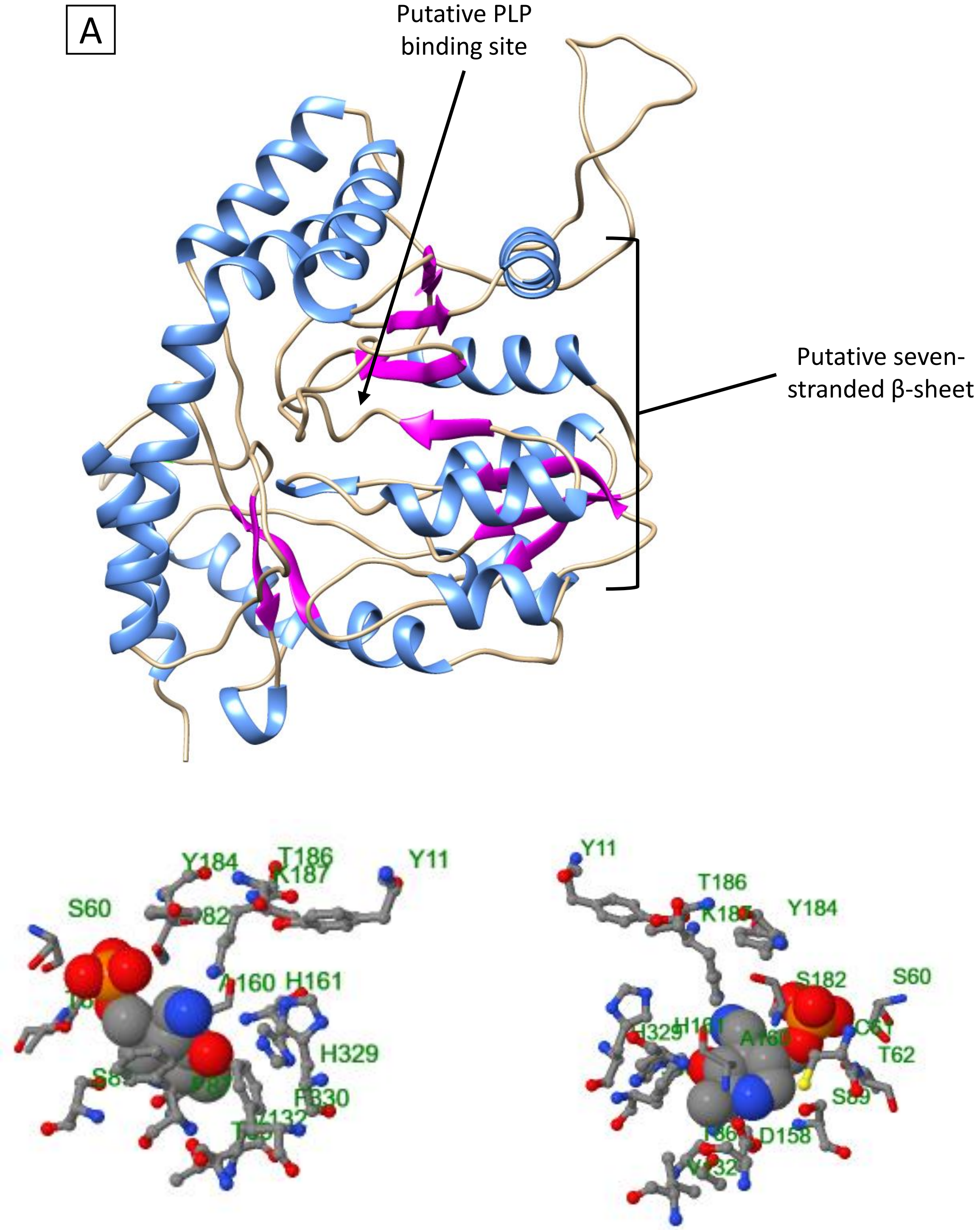

A
Putative PLP binding site
Putative seven-stranded β-sheet
Y184
T186
K187
Y11
S60
H161
H329
Y11
T186
Y184
S182
S60
T62
S89
D158

B

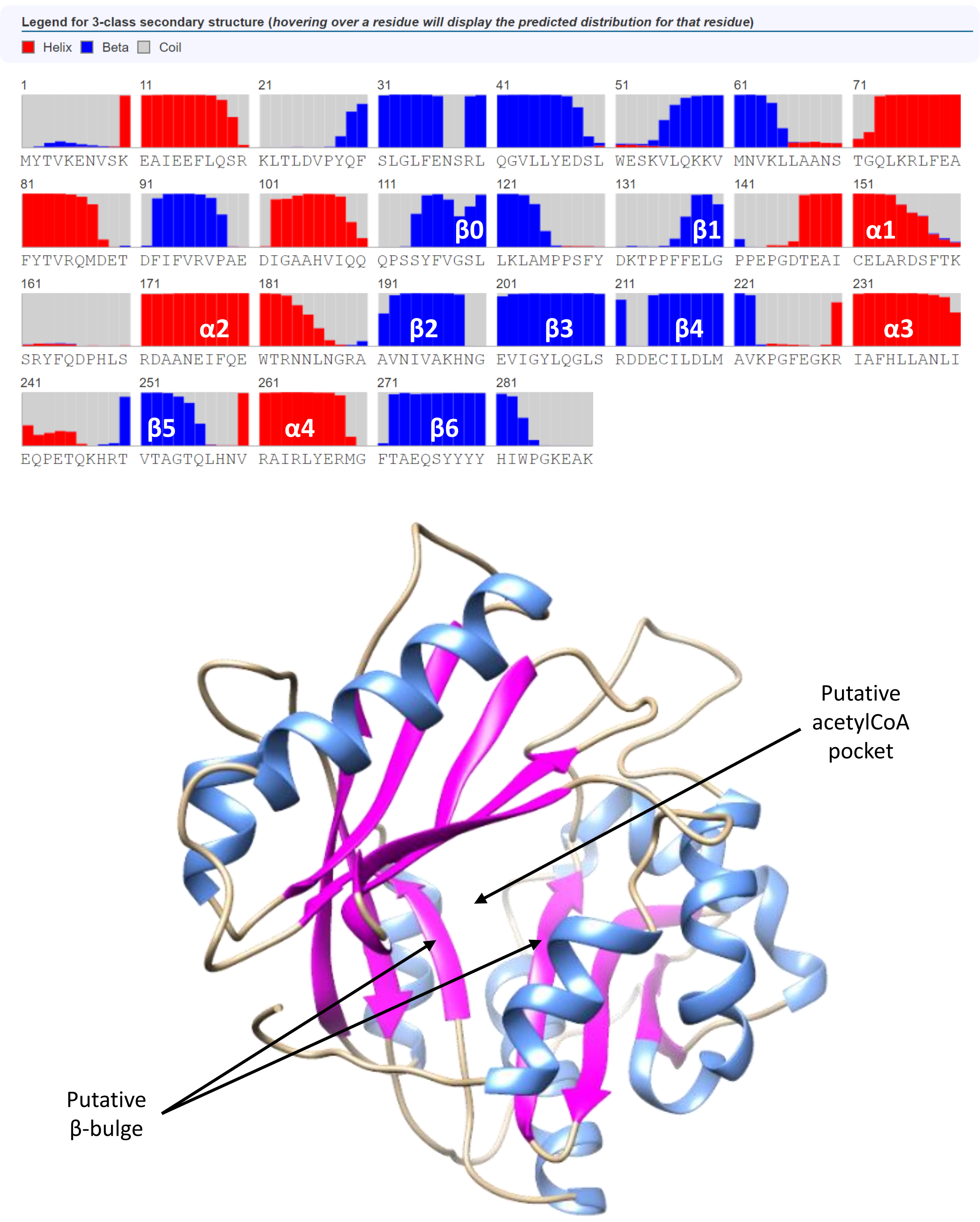
Legend for 3-class secondary structure (hovering over a residue will display the predicted distribution for that residue)
Helix Beta Coil
1 MYTVKENVSK 11 EAIEEFLQSR 21 KLTLDVPYQF 31 SLGLFENSRL 41 QGVLLYEDSL 51 WESKVLQKKV 61 MNVKLLAANS 71 TGQLKRLFEA
81 FYTVRQMDET 91 DFIFVRVPAE 101 DIGAAHVIQQ 111 QPSSYFVGSL 121 LKLAMPPSFY 131 DKTPPFFELG 141 PPEPGDTEAI 151 CELARDSFTK
161 SRYFQDPHLS 171 RDAANEIFQE 181 WTRNNLNGRA 191 AVNIVAKHNG 201 EVIGYLQGLS 211 RDDECILDLM 221 AVKPGFEGKR 231 IAFHLLANLI
241 EQPETQKHRT 251 VTAGTQLHNV 261 RAIRLYERMG 271 FTAEQSYYYY 281 HIWPGKEAK
β0 β1 α1 α2 β2 β3 β4 α3 β5 α4 β6
Putative acetylCoA pocket
Putative β-bulge

C

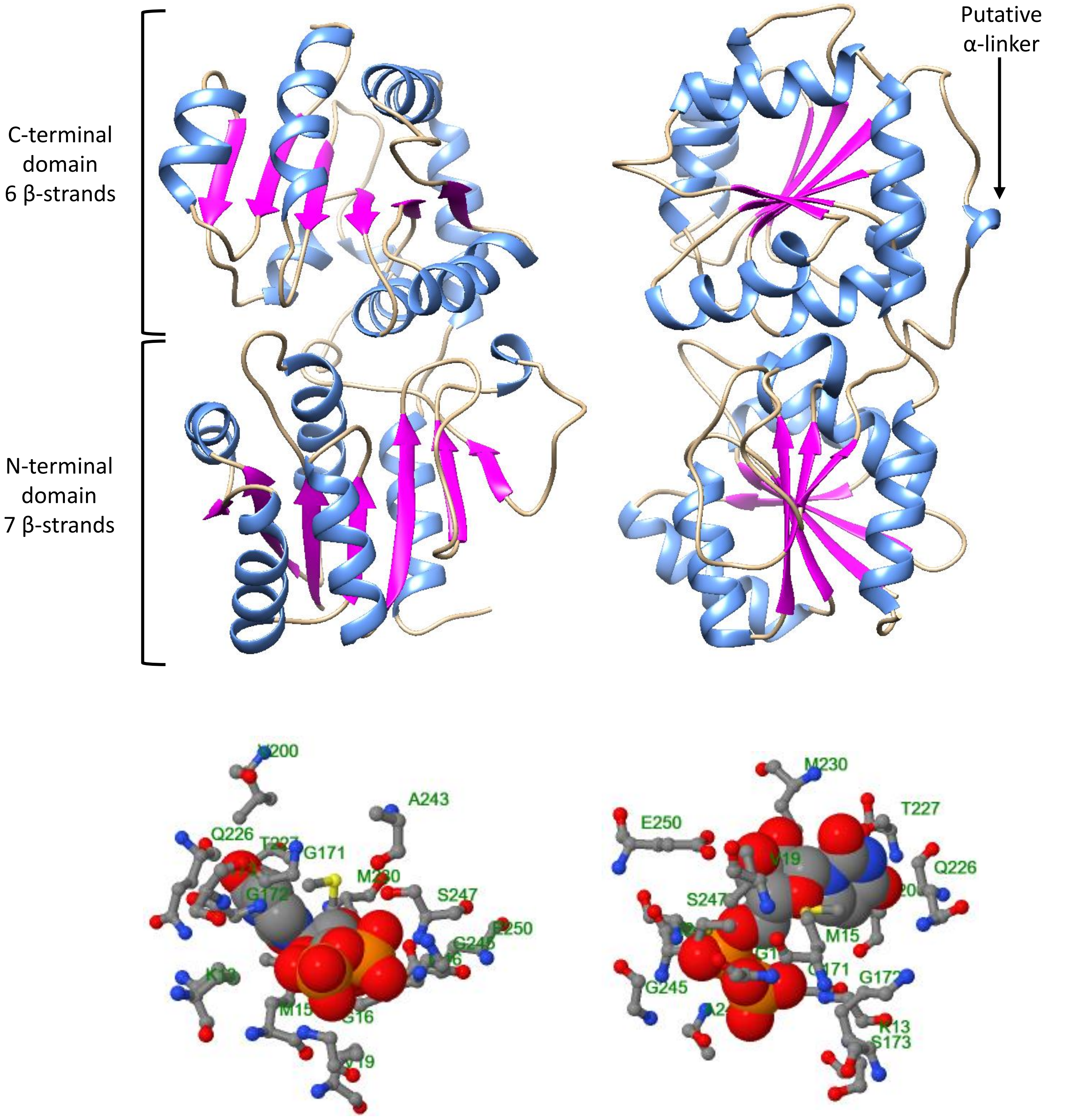

C-terminal domain 6 β-strands
N-terminal domain 7 β-strands
Putative α-linker
A243
Q226
G171
G172
S247
M15
G16
M230
T227
E250
Q226
S247
G245
G172
S173
K13

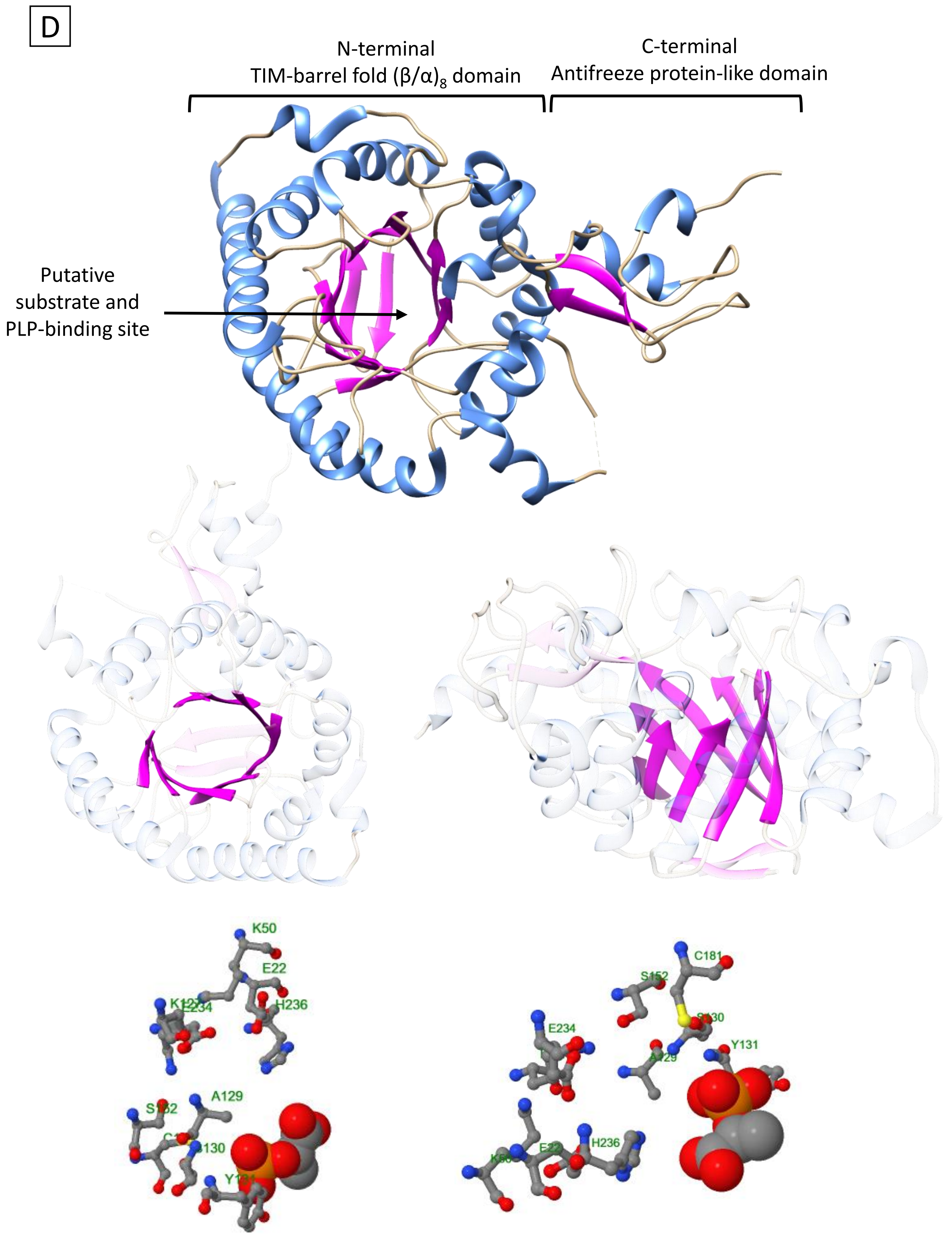
D
N-terminal
TIM-barrel fold (β/α)8 domain
C-terminal
Antifreeze protein-like domain
Putative
substrate and
PLP-binding site
K50
E22
H236
A129
C181
E234
Y131
H236

E

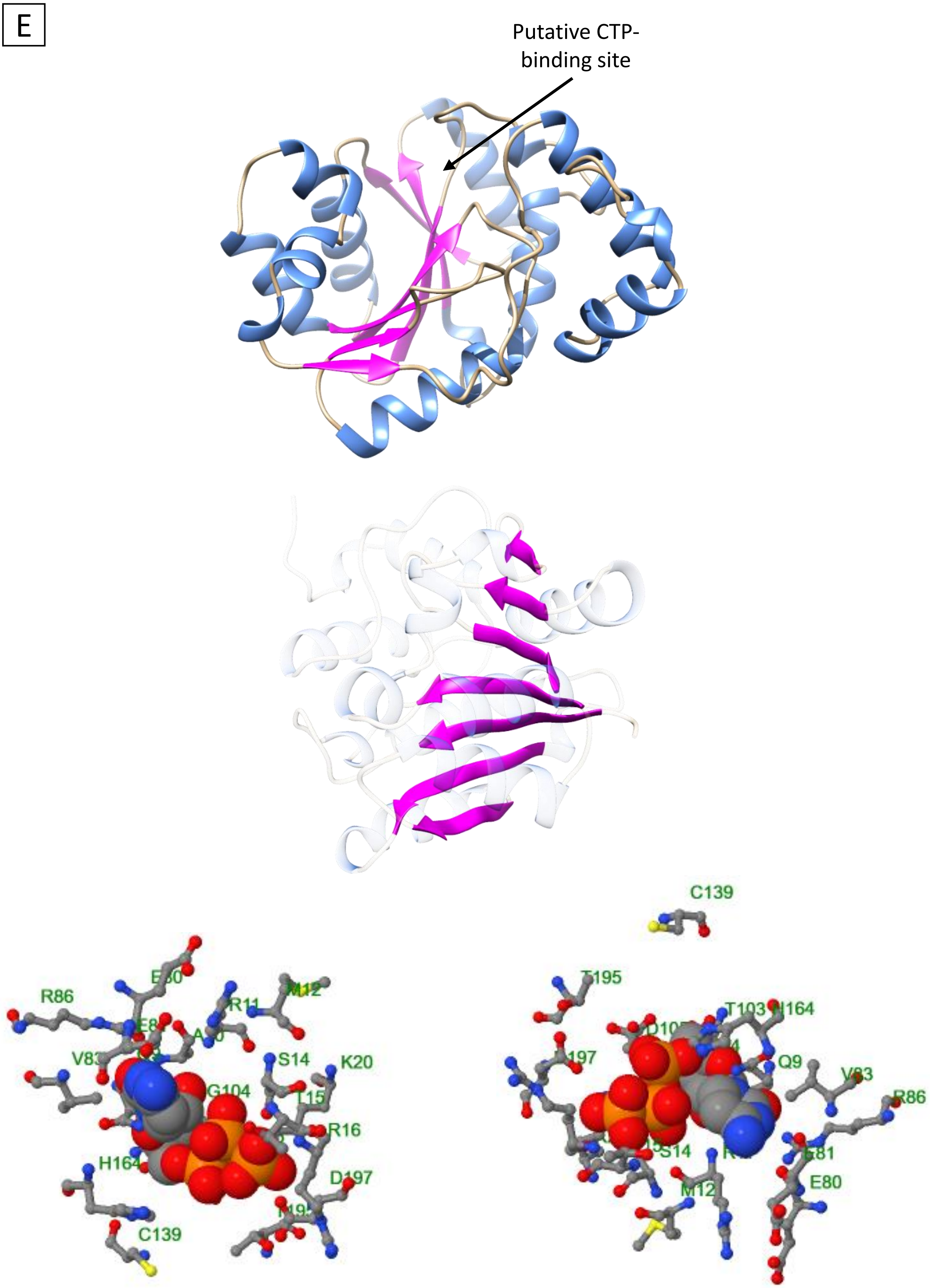
Putative CTP-binding site
R86
M12
R11
V83
S14
K20
G104
T15
R16
H164
D197
C139
C139
T195
T103
H164
Q9
V83
R86
S14
E81
M12
E80
