# Supplementary material for: The sps Genes Encode an Original Legionaminic Acid Pathway Required for Crust Assembly in Bacillus subtilis
Source: mBio. 2020 Aug 18;11(4):e01153-20. doi: 10.1128/mBio.01153-20 (PMC7439481; doi:10.1128/mBio.01153-20)
Supplement: TEXT S2 [file mBio.01153-20-s0002.docx]

The second step of the putative CMP-Leg pathway in *B. subtilis* consists in the transfer of an amino group from the glutamate onto the C4 atom of the UDP-4-keto-6-deoxy-GlcNAc to produce UDP-4-amino-6-deoxy-GlcNAc (**Fig. 3A**). The comparison of the SpsC protein sequence to the TrEMBL database indicates that SpsC is a putative aminotransferase of the DegT family and the RaptorX software identified the structure of the ArnB aminotransferase of *Salmonella typhimurium* as the most related to SpsC (**Table S1**). Interestingly, ArnB catalysis amino group transfer from glutamic acid to the 4"-position of a UDP-linked ketopyranose molecule, which is exactly the presumed activity of SpsC (1). In addition, the predictive structure of SpsC showed similarity with the structure of PseC, the aminotransferase catalyzing the second step of the Pse biosynthetic pathway in *H. pylori* (2). Thus, the predictive structure of SpsC contains a PLP binding pocket and the N-Terminal domain of the protein has a seven-stranded β-sheet arranged in the topological order ↑β1-↓β7-↑β6-↑β5-↑β4-↑β2-↑β3 (3) (**Fig. S6A**). These data make SpsC a good candidate for catalyzing the second step of the Leg biosynthesis pathway in *B. subtilis*. The third step consists in the conversion of the UDP-4-amino-6-deoxy-GlcNAc to UDP-2,4-diNAc-6-deoxy-Glc. In *H. pylori*, the GCN5-related N-Acetyltransferase (GNAT) PseH participating to the CMP-Pse pathway catalyzes the same reaction, but it uses UDP-4-amino-6-deoxy-AltNAc as a substrate (4). The enzymes of the GNAT family have been shown to catalyze the transfer of an acetyl group from acetyl-CoA to the primary amino group of a wide variety of substrates including proteins, mycothiol and aminoglycosides (5). In *B. subtilis*, the *spsD* gene encodes a putative N-Acetyltransferase of the GNAT family. The analysis of the predictive secondary and tertiary structure of SpsD confirms that this enzyme fulfills the characteristics of the GNAT family: a conserved β0- β1-α1-α2-β2-β3-β4-α3-β5-α4-β6 fold and at least one V-like shape named “β-bulge”, that serves as a binding site for acetyl-CoA **(Fig S6B)**. In addition, the enzyme with the most closely related tertiary structure to SpsD is GlmA, a GNAT N-Acetyltransferase catalyzing the transfer of an acetyl group from acetyl CoA to the primary amino group of glucosamine (**Table S1**) (6). Based on these elements, we assume that SpsD catalyzes the third step of the CMP-Leg pathway of *B. subtilis* (**Fig. 3A**). The fourth step of the putative CMP-Leg pathway in *B. subtilis* consists in the hydrolysis of the UDP-2,4-diNAc-6-deoxy-Glc to generate the nucleotide free sugar 2,4-diNAc-6-deoxy-Man. In the Pse biosynthesis pathway of *H. pylori,* the UDP hydrolysis of the UDP-2,4-diNAc-6-deoxy-Alt is catalyzed by the inverting nucleotide sugar hydrolase PseG that belongs to the glycosyltransferase B family (GT-B) (7,8). The structural analysis of PseG showed that this protein has a N-terminal domain harboring seven β-strands in a parallel orientation, a short α-helix linker and a C-terminal domain with six β-strands in a parallel orientation (8). In *B. subtilis*, the *spsG* gene encodes a putative glycosyltransferase with a predicted tertiary structure similar to PseG and harboring a putative UDP binding pocket (**Table S1**, **Fig. S6C**). The UDP-hydrolase activity of SpsG is supported by the RaptorX analysis which identifies MurG, a glycosyltransferase with a UDP-hydrolase activity, as the enzyme the most structurally related to SpsG (7) (**Table S1**). In the CMP-Leg biosynthesis pathway of *C. jejuni*, it has been suggested that the nucleotidase LegG performs a C2 epimerization resulting in NDP removal from the Leg precursor (9). Therefore, we propose that SpsG performed the C2-epimerization and the UDP-hydrolysis of the UDP-2,4-diNAc-6-deoxy-Glc to produced 2,4-diNAc-6-deoxy-Man (**Fig. 3A**)*.* The crystal structure of SpsE was obtained by x-ray diffraction at a resolution of 2.38 Å and it was deposited in the PDB database by the Joint Center for Structural Genomics (unpublished data) (**Fig. S6D**). The protein was crystallized as the apo-enzyme with a divalent cation (Zn^2+^). The SpsE monomer contains a N-terminal triosephosphate isomerase (TIM)-barrel fold domain and a C-terminal antifreeze protein-like domain. The TIM-barrel of SpsE consists of eight α-helices and eight parallel β-strands that alternate along the N-terminal part of the protein forming a cavity that contains a putative PEP-binding domain (**Fig S6D)**. The structure of SpsE is similar to that of NeuB, a sialic acid synthase of *N. meningitidis* that converts phosphoenolpyruvate and N-acetylmannosamine to N-acetylneuraminic (10)*.* Both proteins notably share a C-terminal antifreeze domain and a N-terminal TIM-barrel fold containing a PEP-binding domain and a divalent cation-binding site. These structural data provide a body of evidence suggesting that SpsE catalyzes the condensation between phosphoenolpyruvate and 2,4-diNAc-6-deoxy-Man to generate Leg. To be transferred onto their final acceptor by a specific transferase, nonulosonic acids must be activated with a nucleotide sugar. The *spsF* gene encodes a putative cytidylyltransferase that might play this role in the CMP-Leg pathway of *B. subtilis*. The predictive tertiary structure of the SpsF protein has an αβα three-layer architecture with seven β-strands in the topological order ↑β3-↑β2-↑β1-↑β4-↓β8-↑β5-↑β9 and a putative CTP-binding site, all of which are structural features found in cytidylyltransferase (**Fig. S6E**) (11). In addition, we showed that Leg was not transferred from the mother cell to the surface of the forespore in a ∆*spsF* mutant strain during sporulation, which is the expected outcome for a mutant unable to activate the Leg into CMP-Leg. These data support the hypothesis that SpsF is the cytidylyltransferase of the CMP-Leg pathway in *B. subtilis*.

**References**

1. Noland BW, Newman JM, Hendle J, Badger J, Christopher JA, Tresser J, et al. Structural studies of Salmonella typhimurium ArnB (PmrH) aminotransferase: a 4-amino-4-deoxy-L-arabinose lipopolysaccharide-modifying enzyme. Structure. 2002 Nov 1;10(11):1569–80.

2. Salah Ud-Din AIM, Roujeinikova A. Flagellin glycosylation with pseudaminic acid in Campylobacter and Helicobacter: prospects for development of novel therapeutics. Cell Mol Life Sci. 2018 Apr 27 ;75(7):1163–78.

3. Schoenhofen IC, Lunin V V., Julien JP, Li Y, Ajamian E, Matte A, et al. Structural and functional characterization of PseC, an aminotransferase involved in the biosynthesis of pseudaminic acid, an essential flagellar modification in Helicobacter pylori. J Biol Chem. 2006 Mar 31;281(13):8907–16.

4. Ud-Din AI, Liu YC, Roujeinikova A. Crystal Structure of Helicobacter pylori Pseudaminic Acid Biosynthesis N-Acetyltransferase PseH: Implications for Substrate Specificity and Catalysis. Wlodawer A, editor. PLoS One . 2015 Mar 17;10(3):e0115634.

5. Favrot L, Blanchard JS, Vergnolle O. Bacterial GCN5-Related N-Acetyltransferases: From Resistance to Regulation. Biochemistry. 2016 Feb 23;55(7):989–1002.

6. Reith J, Mayer C. Characterization of a glucosamine/glucosaminide N-acetyltransferase of Clostridium acetobutylicum. J Bacteriol. 2011 Oct;193(19):5393–9.

7. Hu Y, Chen L, Ha S, Gross B, Falcone B, Walker D, et al. Crystal structure of the MurG:UDP-GlcNAc complex reveals common structural principles of a superfamily of glycosyltransferases. Proc Natl Acad Sci U S A. 2003 Feb 4;100(3):845–9.

8. Rangarajan ES, Proteau A, Cui Q, Logan SM, Potetinova Z, Whitfield D, et al. Structural and functional analysis of Campylobacter jejuni PseG. A UDP-sugar hydrolase from the pseudaminic acid biosynthetic pathway. J Biol Chem. 2009;284(31):20989–1000.

9. Schoenhofen IC, Vinogradov E, Whitfield DM, Brisson J-R, Logan SM. The CMP-legionaminic acid pathway in Campylobacter: Biosynthesis involving novel GDP-linked precursors. Glycobiology. 2009;19(7):715–25.

10. Gunawan J, Simard D, Gilbert M, Lovering AL, Wakarchuk WW, Tanner ME, et al. Structural and mechanistic analysis of sialic acid synthase NeuB from Neisseria meningitidis in complex with Mn2+, phosphoenolpyruvate, and N-acetylmannosaminitol. J Biol Chem. 2005 Feb 4;280(5):3555–63.

11. Krapp S, Münster-Kühnel AK, Kaiser JT, Huber R, Tiralongo J, Gerardy-Schahn R, et al. The crystal structure of murine CMP-5-N-acetylneuraminic acid synthetase. J Mol Biol. 2003 Dec 5;334(4):625–37.
