# Supplementary material for: The sps Genes Encode an Original Legionaminic Acid Pathway Required for Crust Assembly in Bacillus subtilis
Source: mBio. 2020 Aug 18;11(4):e01153-20. doi: 10.1128/mBio.01153-20 (PMC7439481; doi:10.1128/mBio.01153-20)
Supplement: TABLE S2 [file mBio.01153-20-st002.docx]

A

| **Primers** | **Sequence** | **Restriction site (underlined)** |
| --- | --- | --- |
| spsABCDEF-1 | CG**GGATCC**GTTGAACCCATTCTTGCTTGA | *Bam*HI |
| spsABCDEF-2 | CACTTTTCCCGGAAGCCTTTTGGAACTTCACACCTGAAACT | - |
| spsABCDEF-3 | AGTTTCAGGTGTGAAGTTCCAAAAGGCTTCCGGGAAAAGTG | - |
| spsABCDEF-4 | ACGC**GTCGAC**TTGGAACTTCACACCTGAAACT | *Sal*I |
| spsA-1 | GACG**CCATGG**AATATGGATAC | *Nco*I |
| spsA-2 | CTTAAATAGGCCGACAAGCTCATCCTACACCTCCTTCTTGGAA | *-* |
| spsA-3 | TTCCAAGAAGGAGGTGTAGGATGAGCTTGTCGGCCTATTTAAG | *-* |
| spsA-4 | CG**GGATCC**CTGAGTGGCAATGAGCACAA | *Bam*HI |
| spsB-1 | CATG**CCATGG**CACCTTATGAGGCTGATGCTT | *Nco*I |
| spsB-2 | TTTCTTTGCGCTCCTATCATTGCTCTCACCCCATTCCAAG | *-* |
| spsB-3 | CTT**GGAATGG**GGTGAGAGCAATGATAGGAGCGCAAAGAAA | *-* |
| spsB-4 | CG**GGATCC**ACTTGCAGAACATATAGATGCCA | *Bam*HI |
| spsD-1 | CATG**CCATGG**GTCATTACCTCTCCGCTTACG | *Nco*I |
| spsD-2 | GCCCGGCCAAATATGATATTAATCAGCCCCTTTTACGAT | *-* |
| spsD-3 | ATCGTAAAAGGGGCTGATTAATATCATATTTGGCCGGGC | *-* |
| spsD-4 | CG**GGATCC**CGAAGCACTGCGATATTGTC | *Bam*HI |
| spsF-1 | CCG**GAATTC**TGTAGGCAAAGACGCTCCTGT | *Eco*RI |
| spsF-2 | CGAAGATCCCAACATGCATGGGAAACGGGCTGTCCTTCAGTA |  |
| spsF-3 | TACTGAAGGACAGCCCGTTTCCCATGCATGTTGGGATCTTCG |  |
| spsF-4 | CG**GGATCC**ATAGCTGTTACCCGTATAATGCCTT | *Bam*HI |
| spsM-1 | CTGA**GAATTC**AACTTAAATTCACG | *Eco*RI |
| spsM-2 | CTAATGGATAGACAGGCTTGTTTAACTCTCGTTTCTCTACCATGC | *-* |
| spsM-3 | GCATGGTAGAGAAACGAGAGTTAAACAAGCCTGTCTATCCATTAG | *-* |
| spsM-4 | CGC**GGATCC**AAGGAGGCACCAGTATGTCTG | *Bam*HI |
| cotE-F | CCG**GAATTC**GAATACAGGGAAATTATTACGAAGGC | *Eco*RI |
| cotE-R | CG**GGATCC**AGTACCATACGTTTATATCATAATACCCTTC | *Bam*HI |
| cotZ-F | CCG**GAATTC**AGCCAGAAAACATCAAGCTG | *Eco*RI |
| cotZ-R | CG**GGATCC**TTATCGAGTTCGCCGACAT | *Bam*HI |
| cotX-F | CCG**GAATTC**CTGACTGTGACCATCCGT | *Eco*RI |
| cotX-R | CG**GGATCC**GCTGTTACAACTGATGTTACG | *Bam*HI |
| spsA-comp-1 | GC**TCTAGA**CAAATCCCTGCTGTCCTTG | *Xba*I |
| spsA-comp-2 | AA**CTGCAG**TCACCCCATTCCAAGCTTT | *Pst*I |
| spsA-comp-3 | CG**GGATCC**CAAATCCCTGCTGTCCTTG | *Bam*HI |
| spsB-comp-1 | G**GAATTC**AACTTCACACCTGAAACTATCGTT | *Eco*RI |
| spsB-comp-2 | G**GAATTC**GGAATGGGGTGAGAGCTTG | *Eco*RI |
| spsB-comp-4 | GG**ACTAGT**TTATTCAGTATCTTCCCGCCTG | *Spe*I |
| spsD-comp-2 | TAATCAGCCCCTTTTACGATATTTGGAACTTCACACCTGAAACT | *-* |
| spsD-comp-3 | AGTTTCAGGTGTGAAGTTCCAAATATCGTAAAAGGGGCTGATTA | *-* |
| spsD-comp-4 | AA**CTGCAG**CATTATTTCGCCTCCTTGC | *Pst*I |
| spsF-comp-1 | G**GAATTC**TGAAGGACAGCCCGTTTC | *Eco*RI |
| spsF-comp-2 | GG**ACTAGT**CTAGTCAGCCTCTCGTTC | *Spe*I |
| spsM-comp-1 | G**GAATTC**AGTGCTTAGCAGCATTGTTTT | *Eco*RI |
| spsM-comp-4 | TA**ACTAGT**TAATAAATAAACTGTTAAAAACAGGCC | *Spe*I |
| spsM-M146A-2 | GATAACAGCTTGGTGGCACC**CGC**TGTGTTCACAGGAGATAC | *-* |
| spsM-M146A-3 | GTATCTCCTGTGAACACA**GCG**GGTGCCACCAAGCTGTTATC | *-* |
| spsM-K150A-2 | CAGCTTTTCAGATAACAG**CGC**GGTGGCACCCATTGTGTTC | *-* |
| spsM-K150A-3 | GAACACAATGGGTGCCACC**GCG**CTGTTATCTGAAAAGCTG | *-* |
| PspsM-F | CCG**GAATTC**GCAGTGCTTAGCAGCATTGTT | *Eco*RI |
| PspsM-R-pen | ACATGAAAATATATACCTCCTCATATTAAACAACTCTCGTTTCTCTACCATGC | - |
| pen-F | GCATGGTAGAGAAACGAGAGTTGTTTAATATGAGGAGGTATATATTTTCATGT | - |
| pen-R | AA**CTGCAG**TCATATTTTCTCCCCTCCTGTTA | *Pst*I |
| PspsM-R-pal | ATTTTCTCCCCTCCTGTTAATAGCCAACTCTCGTTTCTCTACCATGC | - |
| pal-F | GCATGGTAGAGAAACGAGAGTTGGCTATTAACAGGAGGGGAGAAAAT | - |
| pal-R | AA**CTGCAG**ACCATTACTCTTTCTCCTTAGGATTAA | *Pst*I |
| PspsM-R-legB | CAGCACCTGTAACTAAAATATTTCTCACGGCTTATCCTCCAACTCTCGTT |  |
| legB-F | AACGAGAGTTGGAGGATAAGCCGTGAGAAATATTTTAGTTACAGGTGCTG |  |
| legB-R | AA**ACTAGT**TAAACATTATAAAGCTCGCTTTTATAAT | *Spe*I |
| PspsM-R-pglF | GCTAATCTTTTGCTTTTATAAAAAATCATGGCTTATCCTCCAACTCTCGTT |  |
| pglF-F | AACGAGAGTTGGAGGATAAGCCATGATTTTTTATAAAAGCAAAAGATTAGC |  |
| pglF-R | AA**ACTAGT**TATACACCTTCTTTATTGTGTTTAAATTC | *Spe*I |
| PspsM-R-pseB | CGTGATTAAGATATTTTTTTTGTTAAACATGGCTTATCCTCCAACTCTCGTT |  |
| pseB-F | AACGAGAGTTGGAGGATAAGCCATGTTTAACAAAAAAAATATCTTAATCACG |  |
| pseB-R | AA**ACTAGT**TTAAAAACCTTCAGTATGATTGATGAT | *Spe*I |
| PspsA-F | CG**GGATCC**CAAATCCCTGCTGTCCTTG | *Bam*HI |
| PspsA-R | CC**AAGCTT**AACTTCACACCTGAAACTATCGTT | *Hind*III |

B

| **Plasmid name** | **Plasmid features** |
| --- | --- |
| pMADΩ*spsABCDEF* | 5' and 3' regions flanking the *spsABCDEF* operon were amplified by SOE-PCR using primers spsABCDEF-1/spsABCDEF-2 and spsABCDEF-3/spsABCDEF-4. The PY79 chromosomal DNA was used as template. The resulting fragment was purified as a *Bam*HI-*Sal*I fragment and it was inserted between the *Bam*HI and *Sal*I sites of pMAD. |
| pMADΩ*spsA* | 5' and 3' regions flanking the *spsA* gene were amplified by SOE-PCR using primers spsA-1/spsA-2 and spsA-3/spsA-4. The PY79 chromosomal DNA was used as template. The resulting fragment was purified as a *Nco*I-*Bam*HI fragment and it was inserted between the *Nco*I and *Bam*HI sites of pMAD. |
| pMADΩ*spsB* | 5' and 3' regions flanking the *spsB* gene were amplified by SOE-PCR using primers spsB-1/spsB-2 and spsB-3/spsB-4. The PY79 chromosomal DNA was used as template. The resulting fragment was purified as a *Nco*I-*Bam*HI fragment and it was inserted between the *Nco*I and *Bam*HI sites of pMAD. |
| pMADΩ*spsD* | 5' and 3' regions flanking the *spsD* gene were amplified by SOE-PCR using primers spsD-1/spsD-2 and spsD-3/spsD-4. The PY79 chromosomal DNA was used as template. The resulting fragment was purified as a *Nco*I-*Bam*HI fragment and it was inserted between the *Nco*I and *Bam*HI sites of pMAD. |
| pMADΩ*spsF* | 5' and 3' regions flanking the *spsF* gene were amplified by SOE-PCR using primers spsF-1/spsF-2 and spsF-3/spsF-4. The PY79 chromosomal DNA was used as template. The resulting fragment was purified as a *Eco*RI*-Bam*HI fragment and it was inserted between the *Eco*RI and *Bam*HI sites of pMAD. |
| pMADΩ*spsM* | 5' and 3' regions flanking the *spsM* gene were amplified by SOE-PCR using primers spsM-1/spsM-2 and spsM-3/spsM-4. The PY79 chromosomal DNA was used as template. The resulting fragment was purified as a *Eco*RI­-*Bam*HI fragment and it was inserted between the EcoRI and BamHI sites of pMAD. |
| pMUTIN4 *cotE* | An internal fragment of the *cotE* gene was amplified by PCR using primers cotE-F and cotE-R. The PY79 chromosomal DNA was used as template. The resulting fragment was purified as a *Eco*RI*-Bam*HI fragment and it was inserted between the *Eco*RI and *Bam*HI sites of pMUTIN4. |
| pMUTIN4 *cotZ* | An internal fragment of the *cotZ* gene was amplified by PCR using primers cotZ-F and cotZ-R. The PY79 chromosomal DNA was used as template. The resulting fragment was purified as a *Eco*RI*-Bam*HI fragment and it was inserted between the *Eco*RI and *Bam*HI sites of pMUTIN4. |
| pMUTIN4 *cotX* | An internal fragment of the *cotX* gene was amplified by PCR using primers cotX-F and cotX-R. The PY79 chromosomal DNA was used as template. The resulting fragment was purified as a *Eco*RI*-Bam*HI fragment and it was inserted between the *Eco*RI and *Bam*HI sites of pMUTIN4. |
| pBS1CΩ*amyE ::spsA* | The *spsA* gene was amplified with its promoter region using primers spsA-comp-1 and spsA-comp-2 with PY79 chromosomal DNA as template. The resulting fragment was inserted between the *Xba*I and *Pst*I sites of pBS1C. |
| pBS1CΩ*amyE ::spsB* | The promoter region of *spsA* and the *spsB* gene were amplified with the primers spsA-comp-3/spsB-comp-1 and spsB-comp-2/spsB-comp-4 using the PY79 chromosomal DNA as template. The promoter region of *spsA* was purified as a *Bam*HI*-Eco*RI fragment and the *spsB* gene as a *Eco*RI*/Spe*I fragment. Both fragments were then inserted between the *Bam*HI and *Spe*I sites of pBS1C. |
| pBS1CΩ*amyE ::spsD* | The *spsD* gene was amplified with the promoter region of *spsA* by SOE-PCR using primers spsA-comp-1/spsD-comp-2 and spsD-comp-3/spsD-comp-4. The PY79 chromosomal DNA was used as template. The resulting fragment was purified as a *Xba*I-*Pst*I fragment and it was inserted between the *Xba*I and *Pst*I sites of pBS1C. |
| pBS1CΩ*amyE ::spsF* | The promoter region of *spsA* and the *spsF* gene were amplified with primers spsA-comp-3/spsB-comp-1 and spsF-comp-1/spsF-comp-2 using the PY79 chromosomal DNA as template. The promoter region of *spsA* was purified as a *Bam*HI*-Eco*RI fragment and the *spsF* gene as a *Eco*RI*-Spe*I fragment. Both fragments were then inserted between the *Bam*HI and *Spe*I sites of pBS1C. |
| pBS1CΩ*amyE ::spsM* | The *spsM* gene was amplified with its promoter region using primers spsM-comp1/spsM-comp-4 with PY79 chromosomal DNA as template. The resulting fragment was purified as a *Eco*RI*-Spe*I fragment and it was inserted between the *Eco*RI and *Spe*I sites of pBS1C_._ |
| pBS1CΩ*amyE ::spsM**M146A | The M146A mutation was introduced by SOE-PCR using primers spsM-comp-1/spsM-M146A-2 and spsM-M146A-3/spsM-comp-4. The PY79 chromosomal DNA was used as template. The resulting fragment was purified as a *Eco*RI*-Spe*I fragment and it was inserted between the *Eco*RI and *Spe*I sites of pBS1C. |
| pBS1CΩ*amyE ::spsM**K150A | The K150A mutation was introduced by SOE-PCR using primers spsM-comp-1/spsM-K150A-2 and spsM-K150A-3/spsM-comp-4. The PY79 chromosomal DNA was used as template. The resulting fragment was purified as a *Eco*RI*-Spe*I fragment and it was inserted between the *Eco*RI and *Spe*I sites of pBS1C. |
| pBS1CΩ*amyE ::pen* | The *pen* gene was amplified with the promoter region of *spsM* by SOE-PCR using primers PspsM-F/PspsM-R-pen and pen-F/pen-R. The PY79 and the *B. thuringiensis* ATCC35646 chromosomal DNA were respectively used as template. The resulting fragment was purified as a *Eco*RI*-Pst*I fragment and it was inserted between the *Eco*RI and *Pst*I sites of pBS1C. |
| pBS1CΩ*amyE ::pal* | The *pal* gene was amplified with the promoter region of *spsM* by SOE-PCR using primers PspsM-F/PspsM-R-pal and pal-F/pal-R. The PY79 and the *B. thuringiensis* ATCC35646 chromosomal DNA were respectively used as template. The resulting fragment was purified as a *Eco*RI*-Pst*I fragment and it was inserted between the *Eco*RI and *Pst*I sites of pBS1C. |
| pBS1CΩ*amyE ::pen-pal* | The *pen* and *pal* genes were amplified with the promoter region of *spsM* by SOE-PCR using primers PspsM-F/PspsM-R-pen and pen-F/pal-R. The PY79 and the *B. thuringiensis* ATCC35646 chromosomal DNA were respectively used as template. The resulting fragment was purified as a *Eco*RI*-Pst*I fragment and it was inserted between the *Eco*RI and *Pst*I sites of pBS1C. |
| pBS1CΩ*amyE ::legB* | The *pen* and *pal* genes were amplified with the promoter region of *spsM* by SOE-PCR using primers PspsM-F/PspsM-R-legB and legB-F/legB-R. The PY79 and the *C. jejuni* NCTC11168 chromosomal DNA were respectively used as template. The resulting fragment was purified as a *Eco*RI*-Spe*I fragment and it was inserted between the *Eco*RI and *Spe*I sites of pBS1C. |
| pBS1CΩ*amyE ::pglF* | The *pen* and *pal* genes were amplified with the promoter region of *spsM* by SOE-PCR using primers PspsM-F/PspsM-R-pglF and pglF-F/pglF-R. The PY79 and the *C. jejuni* NCTC11168 chromosomal DNA were respectively used as template. The resulting fragment was purified as a *Eco*RI*-Spe*I fragment and it was inserted between the *Eco*RI and *Spe*I sites of pBS1C. |
| pBS1CΩ*amyE ::pseB* | The *pen* and *pal* genes were amplified with the promoter region of *spsM* by SOE-PCR using primers PspsM-F/PspsM-R-pseB and pseB-F/pseB-R. The PY79 and the *C. jejuni* NCTC11168 chromosomal DNA were respectively used as template. The resulting fragment was purified as a *Eco*RI*-Spe*I fragment and it was inserted between the *Eco*RI and *Spe*I sites of pBS1C. |
| pBS1CΩ*amyE ::PspsA-mcherry* | The promoter region of *spsA* gene was amplified with primers PspsA-F /PspsA-R using the PY79 chromosomal DNA as template. The promoterless *mCherry* gene was purified as a *Xba*I-*Pst*I fragment from pSB1C3 *mCherry_Bsu* (BGSC: ECE756). The promoter region of *spsA* was purified as a *Bam*HI*-Hind*III fragment. Both fragments were then respectively inserted between the *Xba*I*-Pst*I and *Bam*HI-*Hind*III sites of pBS1C. |
